# A qualitative focus groups study to identify how pedestrians perceive the arrival of fully autonomous vehicles

**DOI:** 10.3389/fpsyg.2025.1636225

**Published:** 2025-12-16

**Authors:** Lou Schwartz, Guillaume Gronier

**Affiliations:** Luxembourg Institute of Science and Technology, HANDS, Esch-sur-Alzette, Luxembourg

**Keywords:** fully autonomous vehicles, pedestrians, pedestrian safety, vulnearable road users, vehicle design, qualitative methods, acceptance measurement, focus group

## Abstract

This study explores pedestrian perceptions of fully autonomous vehicles and their acceptance. We organized two focus groups in which participants (7 for each) discussed, firstly, their own behavior when crossing the road in front of a human-driven vehicle and the key factors they considered, as perceived benefits and risks of fully autonomous vehicles. Secondly, the factors that could positively influence their own acceptance of fully autonomous vehicles and their preferred interfaces and behaviors (external human-machine interface) for communicating with the fully autonomous vehicles were discussed. Participants highlighted the importance of driver-related cues, the need for clear information on fully autonomous vehicles behavior and the way in which they will be introduced into the automotive landscape, and they indicated their preference for external human-machine interfaces that signal safe crossing. Concerning the external human-machine interfaces, the participants preferred those that projected a green pedestrian crossing onto the road to indicate that the fully autonomous vehicles would wait for the pedestrian to cross. The findings emphasize pedestrians' concerns and preferences to guide the design of future autonomous vehicles.

## 1 Introduction

With the continuous increase in car traffic worldwide, pedestrian safety remains a priority for most countries. Nevertheless, a preliminary report from the American Governors Highway Safety Association (GHSA) estimates that 7508 pedestrians were killed in the United States in 2022, an alarming figure that has not been this high since 1981.^1^

Indeed, the moment in which a pedestrian crosses the road is one of vulnerability due to the serious harm that can be inflicted by a vehicle. When deciding to cross, pedestrians currently take several factors into account, including those based on their environment and the risks involved, such as waiting for the “right moment,” seeking a safer place to cross, or sometimes abandoning the idea altogether, as well as on their personal characteristics (Rasouli and Tsotsos, 2019), see Section 2.1. However, the arrival of fully autonomous vehicles (FAVs) in the near future is expected to change the decision-making context. Now that manufacturers, researchers and governments are working toward this, issues such as societal and environmental impact, acceptance by pedestrians and ways to communicate with pedestrians, as discussed in Section 2.2, are beginning to be raised. For example, several societal and environmental concerns have already been raised. From an ecological perspective, FAVs could generate significant data processing demands and require critical resources for their electronic components, which may increase their environmental footprint (Taiebat et al., 2018; Frieske and Stieler, 2022). At the same time, the absence of traditional non-verbal cues used by pedestrians when interacting with human drivers, such as eye contact or gestures, raises questions about how safe interactions will be achieved in the future (Rasouli and Tsotsos, 2019; Ren et al., 2016). Finally, issues of acceptance remain critical, as pedestrians' trust, perceived safety, and willingness to interact with FAVs will strongly influence their integration into the urban environment (Deb et al., 2017a; Hewitt et al., 2019; Zhang et al., 2023).

To communicate to the pedestrians its actions, intentions, and status, a FAV can not rely on usual social cues [like eye contact and hand gestures (Rasouli et al., 2017)]. So, the envisaged solution is that FAVs rely on an external interface (eHMI), which is a communication system placed on the exterior of the vehicle to convey information to other road users, including pedestrians. Different modalities solutions such as visual, sound, and haptic, can be used.

This research focuses on how pedestrians perceive the arrival of fully autonomous vehicles. And to ascertain the point of view of the general public on these topics, we organized two focus groups with a total of fourteen participants, see Section 4. The focus group participants commented on their own and each other's behavior, as pedestrians, when crossing the road in front of a human-driven vehicle, completing the knowledge we have about the key factors considered by pedestrians when crossing the road in the current situation. They also considered the factors influencing pedestrian acceptance of FAVs and their preferences in terms of interfaces (eHMIs) and behaviors mediated by the eHMI (as signaling intention to stop or to continue driving), see Section 5. The results are discussed in Section 6.

## 2 Related works

The following section presents related works that consider the behavior of pedestrians when crossing the road in front of a human-driven vehicle, that define fully autonomous vehicles and the anticipation of their impact on society, and that propose ways for fully autonomous vehicles and pedestrians to communicate.

### 2.1 Behavior of pedestrians when crossing the road

Much research has focused on observing the behavior of pedestrians when crossing the road, in particular, the explicit, for example, signaling to the driver to stop at the pedestrian crossing, and implicit interactions, e.g., the pedestrian may turn slightly away from the crosswalk to indicate that he does not wish to cross, between pedestrians and drivers of motor vehicles.

#### 2.1.1 Factors considered in road-crossing situations with conventional vehicles

When a pedestrian considers crossing the road in front of a conventional car (driven by a human), all signals transmitted by the driver are important (Rasouli et al., 2017; Sucha et al., 2017). Whether the perceived attention of the driver, gestures made with a hand or the head, flashing headlights or eye contact, all provide indications for pedestrians to decide how safe it is to cross the road. In addition, the approach speed of the vehicle is an important decision-making parameter (Bella and Silvestri, 2015; Liu and Tung, 2014; Rasouli and Tsotsos, 2019; Varhelyi, 1998). According to Rasouli and Tsotsos (2019), when a pedestrian crosses the road, decision-making is also influenced by environmental factors and those inherent to the pedestrian, such as the time of day, perceived distance of the vehicle, and the pedestrian's age, gender and culture (nationality, country of residence, etc.).

#### 2.1.2 Pedestrian behaviors observed in crossing situations

A study by (Sucha et al. 2017) quantifies pedestrian behaviors when crossing a road at a zebra crossing without traffic lights. Of the pedestrians observed, 46% waited until vehicles stopped before crossing; 18% waited until the vehicles slowed down, 2% crossed spontaneously without waiting for a reaction from the vehicles or drivers, and in 34% of observations, the vehicles did not give way at the zebra crossing, forcing the pedestrians to cross after they had passed. Most of those questioned also declared that they tried to signal their intention to cross to the approaching vehicles using a variety of different behaviors: 84% tried to make eye contact with drivers, 9% indicated their intention to cross by stepping into the road, 4% made a hand gesture, 2% thanked the driver, and 1% did nothing.

Another study, this time concentrating on drivers, found that drivers tried to make eye contact with pedestrians to be sure that they had seen the approaching car (Ren et al., 2016). In the absence of eye contact, drivers approached the pedestrian crossing faster and with a less progressive deceleration than when eye contact is made. Eye contact is thus perceived by the driver as the pedestrian expressing his/her intention to cross, thus inciting the driver to better anticipate stopping. Lee et al. highlighted that less than 1% of the vehicles gave pedestrians an indication that they were letting them cross, for example, by flashing their headlights or sounding their horn (Lee et al., 2021). However, approximately 4% of drivers made a hand gesture when they let pedestrians cross.

#### 2.1.3 The decision-making process for crossing the road

According to Ezzati Amini et al. (2019), the vehicle-pedestrian interaction can be divided into five different phases, resumed in an interaction process: (1) before any interaction, the interacting parties quickly assess the characteristics of the road in the approach to the contact zone. This is the moment in which the pedestrians choose their crossing point while drivers continually monitor the road and traffic and adapt their driving accordingly, (2) then, the pedestrians implicitly or explicitly indicate their intention to cross, (3) the interaction as such begins at the moment in which the parties mutually detect each other, both parties (4) assess the situation according to the information at their disposal, and (5) choose a strategy.

Liu et al. (2021) propose a cognitive decision-making and behavioral model for crossing the road made up of three parts: situational awareness, risk assessment, based on the perception of the hazard, and decision-making, based on the personal acceptance of the risk. A pedestrian's situational awareness includes his/her ability to perceive objects in their environment (perception), understand the state and intention of these objects (understanding) and predict the future state of these objects (projection) (Endsley, 2017; Cœugnet et al., 2019). Consequently, pedestrians sense the hazards according to their perception and assess the degree of subjective risk, then decide on the behavior to adopt by comparing the subjective risk with their level of acceptable risk.

These studies thus indicate that a pedestrian-vehicle interaction, whether explicit or implicit, is initiated when pedestrians seek to cross the road. Certain driver behaviors, including eye contact and the visual attention they pay to the environment, enable them to indicate to the pedestrians whether they can cross safely.

As vehicle automation increases, pedestrians are less likely to rely on traditional non-verbal cues like eye contact. Even when a human is physically present in an autonomous vehicle, pedestrians tend to stop relying on eye contact with the passive occupant (Sahaï et al., 2022). Thus, FAVs, whether or not a human is inside, highlight the need for new forms of vehicle–pedestrian interactions that must be devised and implemented. Alternative solutions have been proposed by certain manufacturers and researchers, and we will introduce some of these in the next section.

### 2.2 Fully autonomous vehicles

#### 2.2.1 Definition of a fully autonomous vehicle

Manufacturers are carrying out extensive research in order to make vehicles fully autonomous (FAVs).FAVs may be adopted by 24.8%–87.2% of industries by 2045 (Yao et al., 2023). Indeed, road tests are already being carried out by different automobile manufacturers (Bagloee et al., 2016). Numerous governments support research and development into FAVs, not only from an economic point of view, but also by preparing the massive infrastructure adaptations that will be necessary for FAVs and non-autonomous vehicles to share the road (Alsalman et al., 2021).

In Fully Autonomous Vehicles, driving is managed entirely by an automated system, which is equipped with sensors and capable of reacting to all situations the vehicle finds itself in. The US Department of Transportation's National Highway Traffic Safety Administration (NHTSA) defines autonomous vehicles as follows: “*Automated vehicles are those in which at least some aspects of a safety-critical control function (e.g., steering, throttle, or braking) occur without direct driver input*.”^2^

**Figure 1:**
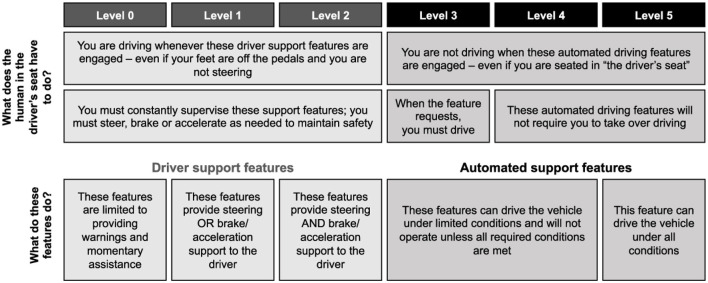
Levels of driving automation, adapted from the SAE taxonomy and definitions for terms related to driving automation systems for on-road motor vehicles - J3016 SAE https://www.sae.org/blog/sae-j3016-update.

FAVs have the highest level of driving automation according to the Society of Automotive Engineers (SAE), which distinguishes 6 levels, from 0 to 5, in the Taxonomy and Definitions for Terms Related to Driving Automation Systems for On-Road Motor Vehicles, see Figure 1.^3^ The first three levels (0, 1, and 2) are considered driving aids, where the driver remains the main operator of the vehicle. The next 3 levels (3, 4, and 5) delegate some or all of the driving to the automated system. FAVs correspond to level 5, where these features can drive the vehicle everywhere and under all conditions.

#### 2.2.2 Societal and environmental impact of FAVs

**Figure 2:**
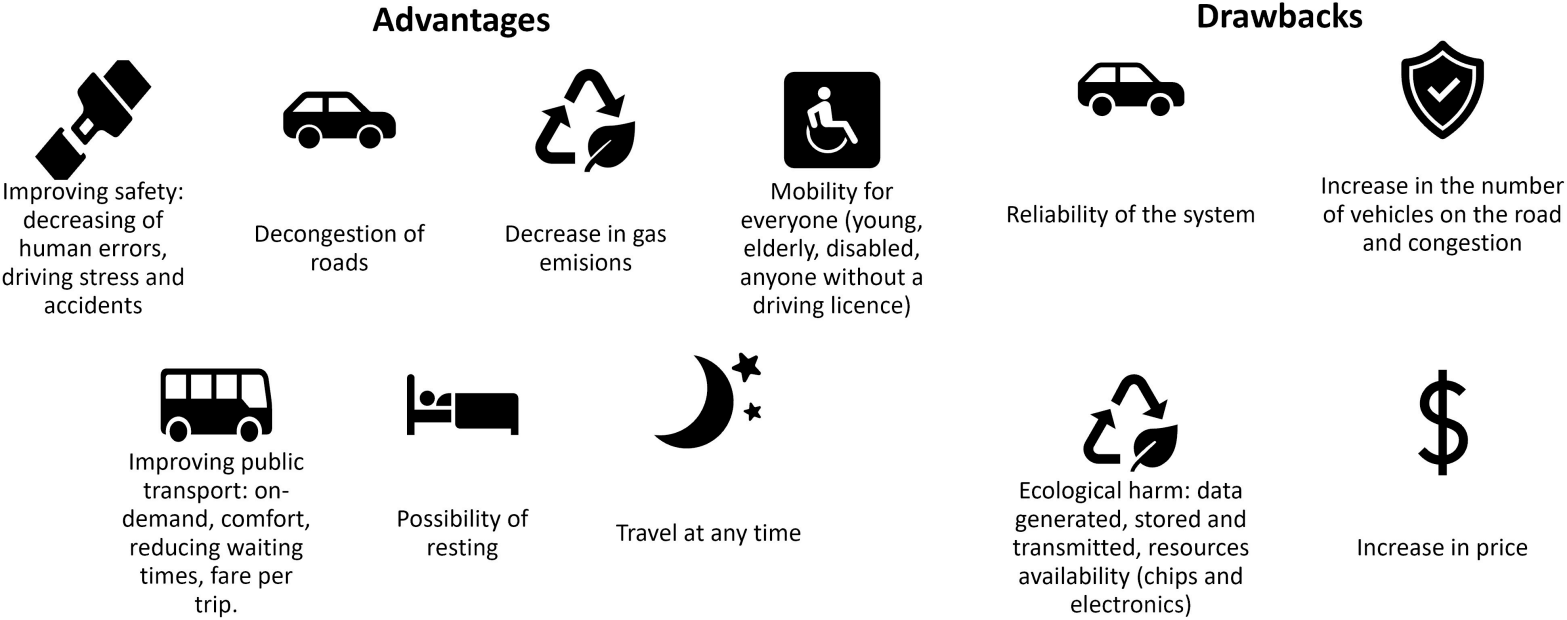
Advantages and drawbacks of FAVs from the state of the art.

FAVs have been the focus of much attention since the beginning of the century due to the numerous changes they promise, both social and in the transport domain, see Figure 2. They pledge to (1) considerably improve road safety by reducing human error (Imprialou and Quddus, 2019); (2) decongest roads, in particular, with the adoption of new mobility options, such as car-sharing, and a reduction in the number of accidents (Bagloee et al., 2016; Lu et al., 2020; Minelli et al., 2015; Tu et al., 2019); and (3) reduce greenhouse gas emissions (estimated reduction from 94% to 87%) thanks to the combination of several factors related to FAVs: smaller vehicle size, optimized road journeys, the favoring of car-sharing, optimal ecological driving and the use of electrical rather than thermal energy (Greenblatt and Saxena, 2015; Jones and Leibowicz, 2019; Pavone, 2015; Sanguesa et al., 2021; Shiwakoti et al., 2020).

The main perceived advantages of FAVs are: a reduction in driving-related stress; the opportunity to relax during long journeys and to spend time on other leisure activities; a reduction in the number of accidents and therefore, the preservation of the physical health of users; a reduction in insurance costs; and the possibility of traveling at any time, even when users are not in a state to drive safely (Pettigrew, 2017; Pettigrew et al., 2018; Pettigrew and Cronin, 2019; Thomopoulos and Givoni, 2015).

FAVs are also viewed as a benefit for public transport by reducing waiting times and fares per trip (Tirachini Hernández and Antoniou, 2020). Pettigrew (2017) argues that the deployment of FAVs would permit improved mobility for young people, the elderly and people with a disability (Pettigrew, 2017). However, it must be pointed out that this high demand from a large population that currently does not have access to individual transport could also have a rebound effect: the number of vehicles on roads would increase significantly, in turn contributing to an increase in traffic congestion, thus having a negative impact (Bissell et al., 2020; Hsu, 2012; Metz, 2018; Minelli et al., 2015).

From an ecological point of view, FAVs could have a harmful effect on the environment due to the increasing amount of data generated (Zanellato and Gombault, 2019). Indeed, FAVs need communication and data processing, which require computational resources and large-scale infrastructures that are energy-intensive and have an environmental impact (Taiebat et al., 2018). Furthermore, current shortages in the resources used for the electrical components of FAVs show that their availability needs to be considered alongside the ecological impact of their use (Frieske and Stieler, 2022).

Finally, questions have been raised about the reliability of the system and its higher costs (Zanellato and Gombault, 2019).

Regardless of the predictions, all the research agrees that the introduction of FAVs will require a thorough and global overhaul of transport methods. Furthermore, the anticipated advantages cannot be effective unless a new global policy is put in place by the authorities (Zanellato and Gombault, 2019).

#### 2.2.3 Acceptance of FAVs by pedestrians

While in the current context (with cars driven by humans), (Deb et al. 2017b) note that a high percentage of pedestrians (around 60%) do not have confidence that drivers would respond appropriately toward them, interactions between pedestrians and FAVs raise new research questions. Indeed, with FAVs, the figures are more ambiguous, since on one hand they are seen as machines that are capable of avoiding human error, while on the other hand, they must be accepted by pedestrians. This acceptance process has been documented in technology acceptance models, such as the Technology Acceptance Model (TAM) (Davis et al., 1989), its extension TAM2 (Venkatesh and Davis, 2000), and the Unified Theory of Acceptance and Use of Technology (UTAUT) (Venkatesh et al., 2003). TAM highlights two central determinants of adoption: perceived usefulness, which refers to the degree to which a person believes a technology will enhance their performance, and perceived ease of use, which corresponds to the degree to which it is seen as effortless (Davis et al., 1989). TAM2 extends this framework by integrating social influence, such as subjective norms and image, as well as cognitive instrumental processes, such as job relevance and output quality (Venkatesh and Davis, 2000). UTAUT synthesizes several prior models and identifies four main constructs that predict behavioral intention and use: performance expectancy, effort expectancy, social influence, and facilitating conditions (Venkatesh et al., 2003). Although these models were originally developed for interactive systems, they have recently been applied to the study of autonomous vehicles, including pedestrians' acceptance (Farzin et al., 2023; Hewitt et al., 2019; Koul and Eydgahi, 2018). They provide a theoretical foundation to understand how perceptions of usefulness, ease of use, and social influence can be translated into attitudes toward FAVs. Building on this framework, several studies have identified multiple factors that specifically influence pedestrian acceptance of FAVs, including general attitudes toward FAVs, social norms, trust, perceived effectiveness, perceived compatibility with the infrastructure, perceived effectiveness of the system, and perceived anxiety and stress (Deb et al., 2017a; Hewitt et al., 2019).

Koul and Eydgahi (2018) aimed to enrich the TAM to evaluate the intention-to-use of driverless car technology (DCT). In addition to the perceived usefulness and perceived ease of use variables, the authors added age and years of driver experience variables. Their results showed that the older and more experienced the subjects, the lower their intention to use FAVs. In another study, Farzin et al. (2023) enhanced the UTAUT model with three variables relating to the adoption of autonomous vehicles: perceived risk, trialability, and observability. This acceptance model indicates that people would feel ready to use autonomous vehicles if they could observe how they worked and learn from the experience of others (observability criterion), if they could try them out long enough to form an opinion (trialability criterion), and if the perceived risks were low enough.

In a systematic review of the literature on acceptance models for FAVs, (Zhang et al. 2023) identified six main acceptance factors. (1) Trust - Studies have shown that trust influences both general acceptance of and intention to use FAVs. Additionally, public trust acts as a mediator between perceived value and acceptance. Individuals with limited driving experience, knowledge of FAVs, and no knowledge of accidents involving FAVs tend to have higher levels of trust in FAV technology. Overall, trust plays a crucial role in shaping perceptions and intentions regarding FAV adoption. (2) Cost - the cost of use particularly affects acceptance by the public and their intention to use FAVs, especially in the context of autonomous public transport and shared FAVs. Additionally, individuals are sensitive to the purchase costs of privately used FAVs, prioritizing them over vehicle attributes. (3) Safety - perceptions of FAV safety impact purchase intentions, while concerns about potential traffic safety degradation and a reluctance to allow children in FAVs contribute to negative attitudes. (4) Age also plays a crucial role in FAV acceptance. The younger demographic tends to maintain a positive attitude toward FAVs, while older individuals may change their perceptions over time. Additionally, the anchoring effect is weaker in individuals aged 42 to 59, making them more open to accepting FAVs in general contexts. (5) Perceived Usefulness (PU) and Perceived Ease of Use (PEOU) also significantly impact FAV acceptance. While both PU and PEOU positively affect attitudes and intentions to use FAVs, PEOU tends to have a stronger influence than PU. These constructs jointly explain a significant portion of behavioral intention, with PU enhancing the perceived benefits and PEOU reducing the perceived costs of FAVs. (6) Finally, various interesting factors influence FAV acceptance. Individuals without a driving license typically oppose shared FAVs without human drivers, while those with a license may either be attracted to or dislike FAVs. Moreover, people who enjoy driving cars tend to have negative attitudes toward FAVs. Conversely, frequent long-distance travelers show enthusiasm for FAVs.

To improve acceptance of FAVs, pedestrians need to communicate with them. One way of doing this is to integrate an external interface (eHMI) on to the vehicle that will be visible to road users (Ackermann et al., 2019). An eHMI refers to a set of solutions (interfaces on FAVs) that could help road users to feel safe by clearly communicating awareness (person and objects detected by the vehicle) (Alhawiti et al., 2024; Schieben et al., 2018), intention (what it is about to do as yielding/not yielding) (Alhawiti et al., 2024; Schieben et al., 2018), status (the current operational mode, e.g., autonomous/manual mode) (Alhawiti et al., 2024; Schieben et al., 2018), and cooperation capabilities (asking other roads users to adopt a behavior like stop or pass) (Schieben et al., 2018) or other information (Alhawiti et al., 2024). Another method to enable interaction between FAVs and pedestrians is to adapt the infrastructure in order to offer a secure environment to users (Bonneviot et al., 2021, 2023).

#### 2.2.4 Communication via an external human machine interface

In certain situations, vehicles need to transmit information to pedestrians, in particular, when pedestrians wish to cross the road. Pedestrians and other vulnerable road users expect an external and explicit communication (Harkin et al., 2024). Several eHMI concepts are proposed by manufacturers and researchers to explore different signaling methods and their understanding and acceptability by pedestrians (Alvarez et al., 2020; Bazilinskyy et al., 2019; Chang et al., 2017; Dey et al., 2018, 2020b; Eisma et al., 2019; Faas and Baumann, 2019; Faas et al., 2020; Li et al., 2018; Liu et al., 2021; Media, 2017; Schwartz et al., 2022). ^4^ ^5^ ^6^ ^7^ ^8^

Several types of eHMI were identified by (Dey et al. 2020a) in a study of literature and prototypes developed by manufacturers, comprising visual, acoustic and haptic interfaces, and using body language artifacts and other eHMIs that do not fall into any of the preceding categories. However, the authors highlight that visual interfaces represent 97% of the eHMIs identified, with 69% using only the visual method and 29% combining visual with another method, such as sound or haptic.

As shown in the literature review, the eHMIs designed to date are very different both in terms of interactions and types of information communicated. There does not currently seem to be a consensus, despite visual eHMIs being the most numerous.

#### 2.2.5 Critical appraisal of previous research

Although the body of literature on pedestrian behavior and the anticipated impact of FAVs is rich, several inconsistencies and methodological limitations deserve mention. Research on pedestrian crossing behavior, for example, has produced mixed results. While some studies emphasize the importance of social cues such as eye contact in guiding decisions (Ren et al., 2016), others report that such cues are rarely used in everyday interactions (Lee et al., 2021). Moreover, this line of research often relies on simulated environments or is limited to specific cultural contexts, which raises questions about the generalizability of the findings (Sucha et al., 2017; Rasouli and Tsotsos, 2019).

When it comes to the definition and anticipated deployment of FAVs, several projections appear overly optimistic. Earlier forecasts predicted widespread deployment within the next decade (Nieuwenhuijsen et al., 2018), yet more recent analyses suggest slower and less predictable adoption.

Regarding the societal and environmental impact of FAVs, the literature is equally divided. Some authors highlight potential ecological benefits through improved efficiency and reduced emissions (Greenblatt and Saxena, 2015; Jones and Leibowicz, 2019), while others warn of rebound effects and the environmental costs of energy-intensive data infrastructures (Taiebat et al., 2018; Bissell et al., 2020).

In the field of acceptance research, the application of technology acceptance models such as TAM or UTAUT has generated useful insights, but it remains problematic to directly transpose frameworks developed for technology users to pedestrians, who are not the primary users of these systems.

Findings are also inconsistent regarding demographic influences, with some studies reporting a lower willingness to adopt FAVs among older adults (Koul and Eydgahi, 2018), while others point to more complex trajectories of acceptance (Zhang et al., 2023). Finally, studies on external human–machine interfaces (eHMIs) reveal a lack of consensus on the most effective solutions. Visual displays dominate the literature, but their inclusivity is contested, and most evaluations have been conducted with prototypes or in laboratory settings rather than real-world contexts (Dey et al., 2018; Liu et al., 2021).

By acknowledging these inconsistencies and methodological constraints, our study aims to complement the literature by providing qualitative insights from pedestrians themselves, thus contributing to a more grounded understanding of their concerns and expectations.

## 3 Objectives and research questions

The promise of the arrival of FAVs has raised questions about their acceptance by the public in general and pedestrians in particular. Our study involved two sets of participants, comprising 14 people in total. Each set was composed of a group of seven individuals who took part in both focus group sessions. The first focus group was dedicated to acceptance factors of FAVs, and the second to preferences for eHMIs. This procedure was repeated with two different groups of participants, thereby broadening the diversity of perspectives. A detailed description of the procedure and protocol is provided in Section 4.1.3.

Building on these discussions, the study seeks to answer the following general research question: **How do pedestrians perceive the arrival of fully autonomous vehicles?** This question can be broken down into four more operational research questions:

- RQ1: What are the key factors considered by pedestrians when crossing the road today?
- RQ2: What are the perceived benefits and risks or problems of FAVs?
- RQ3: What could positively influence the acceptance of FAVs by pedestrians?
- RQ4: How can communication take place between FAVs and pedestrians wishing to cross the road?

## 4 Materials and methods

### 4.1 Procedure

#### 4.1.1 Recruitment survey and questionnaires

Participants were recruited via social networks through online advertisements or email. We worked with a convenience sample, obtained without any particular selection method. As part of the call for participation, candidates were invited to complete an online survey designed to collect background information and characterize their profiles.

This survey included two standardized questionnaires. The Pedestrian Behavior Questionnaire (PBQ) (Deb et al., 2017b) measures the frequency of risky behaviors among pedestrians. It consists of 20 items rated on a 6-point Likert scale (1 = very infrequently to 6 = very often) and requires approximately 5 min to complete. The Pedestrian Receptivity Questionnaire for FAVs (PRQF) (Deb et al., 2017a) assesses pedestrians' willingness to cross the road in front of a fully autonomous vehicle in various situations. It contains 16 items, rated on a 7-point Likert scale (1 = strongly disagree, 7 = strongly agree), and can be completed in about 4 to 5 min.

Both instruments were administered in French. The PRQF underwent a psychometric validation process, which has been published by Gronier et al. (2022). The PBQ was translated into French following the same procedure as the PRQF, ensuring linguistic and conceptual equivalence. While the psychometric validation of the French PBQ is still ongoing, the French items are available in Gronier et al. (2022).

In addition, the survey included sociodemographic questions covering gender, country of residence, age, dependent child(ren) under 10 years old, living environment, travel habits (car, foot, bus, train, etc.), walking time per day and walking frequency per day, as well as whether the participant had difficulty walking, had been the victim of a road accident as a pedestrian or driver, and whether the participant was in possession of a driving license. Overall, participants needed approximately 20 min to complete the full survey.

#### 4.1.2 Focus group methodology

We chose to respond to these hypotheses using the focus group method Cyr, (2019), as it seems to be the most appropriate for investigating the sensitive topic of FAV acceptance. This sensitivity stems from the fact that acceptance of FAVs touches upon issues of safety, trust in technology, and risk perception, which can provoke anxiety and strong opinions among pedestrians. Discussing such concerns in an interactive group setting enables participants to express both fears and expectations, while benefiting from the collective dynamic to elaborate and confront their views. Focus groups serve as a qualitative research approach enabling participants to articulate, elucidate and deliberate on their individual perspectives within an interactive environment. This method facilitates the identification of shared and divergent viewpoints, as well as the exploration of underlying perceptions through collaborative discussion. (Cyr 2019) underlines that focus groups are well-suited to studying socially constructed phenomena due to their ability to capture context and nuance. Participants engage in open discussions, revealing disagreements, contradictions, and the potential for consensus. These phenomena are complex and require a high cognitive effort, often leading individuals to seek shortcuts when grappling with difficult ideas alone. By allowing multiple individuals to navigate complex concepts collectively, focus groups prove more effective than one-on-one interviews or surveys in addressing these challenges. Moreover, focus groups provide a platform for individuals to engage in open communication and are particularly valuable for discussing sensitive or difficult topics. Finally, focus groups serve as valuable tools for researchers exploring new research areas, particularly when there is limited existing knowledge. Participants play a crucial role in uncovering important aspects of a research question, empowering them to control the discussion. By allowing participants to guide the conversation, researchers can identify significant aspects of a new topic, leveraging participant empowerment to their advantage.

#### 4.1.3 Organization of the focus groups

**Figure 3:**
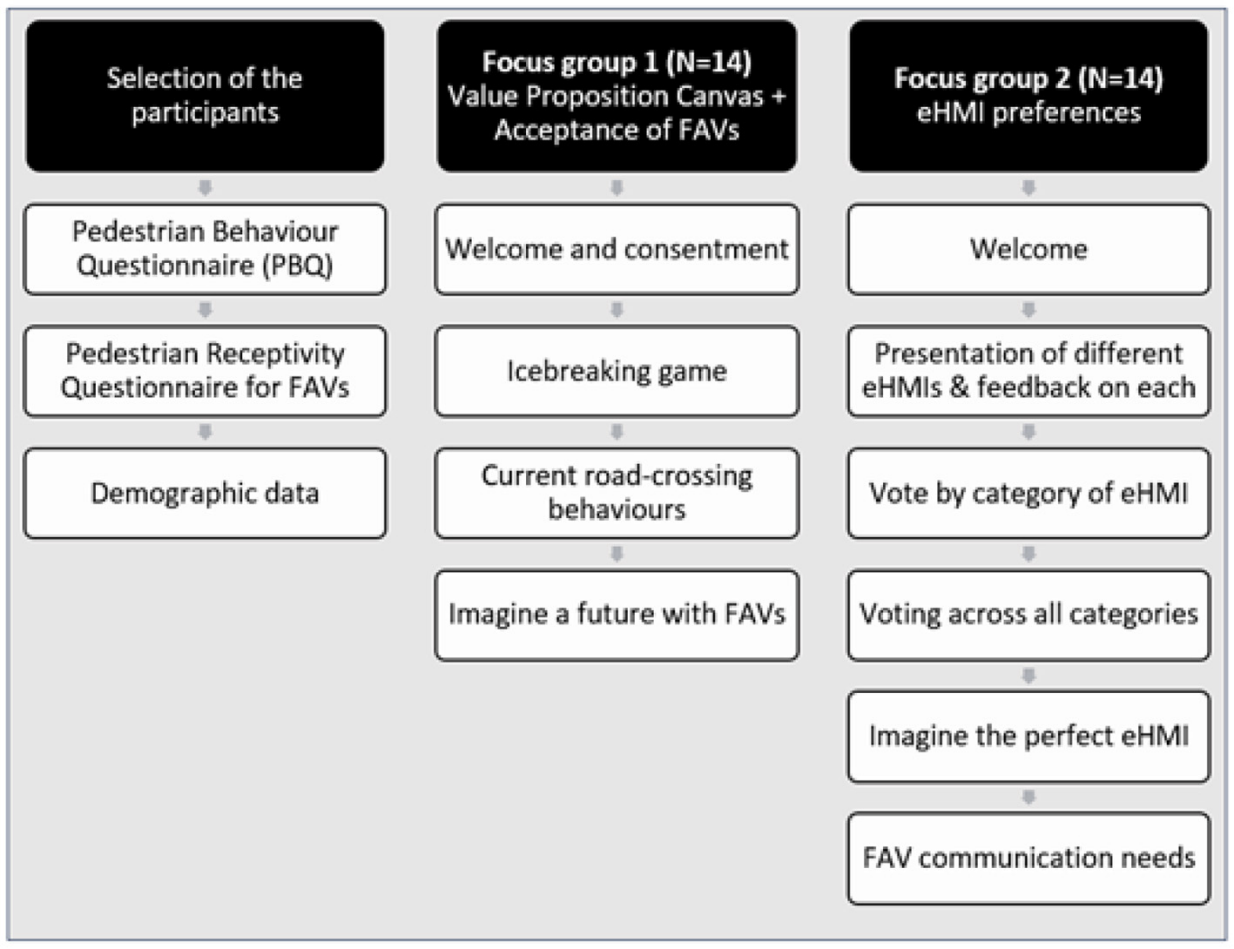
Protocol of the study.

To answer the research questions, we created two focus groups. The purpose of these focus groups was to better understand the issues of pedestrians in crossing situations when a vehicle is approaching, to list some of the factors influencing the adoption of FAVs by the public, and to identify safer and more comprehensive eHMIs. Thus, the first focus group was dedicated to the acceptance factors of FAVs, and the second to the eHMI preferences (see Figure 3 and Annexes for the complete animation guide. Section 11.1). Focus groups rely on group dynamics, allowing for more extensive information to be gathered in short time through social interactions within the group than through individual interviews (Rabiee, 2004). They highlight both personal opinions and differences within the group. However, the quality, diversity, and quantity of the data collected depends on group interactions, and therefore on whether participants feel comfortable with each other and participate equally in discussions. Participant selection is therefore crucial and can influence the results. the presence of a dominant participant can influence the data. As we wanted to involve as many pedestrian profiles as possible, we recruited fourteen participants. However, in order to maintain a good group dynamic and facilitate exchanges, the fourteen participants were split into two groups of seven people each. We ensure us that all selected participants are comfortable to talk in French, even if it is not their native language, by asking them to self-evaluate their French level in the selection questionnaire. The focus group discussions took place in April 2021.

In the following subsections, we will present the topics addressed, the participants and the selection process, the data analysis, and the consideration of ethical and legal issues.

### 4.2 Data collection

The focus groups were organized into two-hour sessions. They were conducted online using Microsoft Teams,^9^ a real time communication tool used as a video conference link, for communication and^10^ to support the interactions and notes. The complete animation guide is available in the annexes. Miro is an online tool to manage teamwork. We used the possibility to share a whiteboard. The whiteboard was set with the different activities' zones and eHMIs images. The participants were able to add notes, comments or votes. Miro also supports collaborative mind mapping.

This research complied with the American Psychological Association Code of Ethics. Informed consent was obtained from each participant.

#### 4.2.1 First focus group: present and future behavior of pedestrians

In the first focus group, the Value Proposition Canvas method was used to identify the main activities of pedestrians when crossing the road in the current context, i.e., without FAVs, the difficulties they encountered, and favorable elements for this activity (Osterwalder et al., 2015). This method is a design tool that structures user experience around three components: (1) activities (“jobs”) performed by the user, (2) difficulties or risks (“pains”) encountered in carrying them out, and (3) beneficial or facilitating aspects (“gains”) that support successful performance. Applied to our study, it enabled participants to systematically reflect on their daily crossing behaviors, the obstacles they face, and the factors that help them feel safe. This structured group activity facilitated the transition to discussions about how FAVs might change these activities and how eHMI could support safer interactions.

Participants were asked to react to a hypothetical scenario of FAVs arriving in their town the following year. Then, they were asked to assimilate the role of pedestrians, in order to comment on the main benefits and risks of FAVs in their opinion, as well as to indicate what might help them consider FAVs reliable vehicles. This focus group raised some ideas for communication between FAVs and pedestrians.

#### 4.2.2 Second focus group: eHMI preferences

The second focus group considered interactions between FAVs and pedestrians. Twenty eHMIs grouped into six categories (textual, illuminated, icons, with road projection, anthropomorphic, other) were presented to the participants along with an image and an explanation of their behavior for each one. For each eHMI listed, participants proposed adjectives to qualify it, suggested improvements and voted twice, for both the most understandable and safest one.

The eHMIs were chosen as follows. Starting from Dey et al. (2020a)'s taxonomy, which distinguishes between textual, illuminated, icon-based, projection-based, anthropomorphic, and other interfaces, we selected representative examples for each category. We deliberately included both academic prototypes and industrial concepts documented in the literature, ensuring that the set covered the diversity of design strategies explored so far (e.g., visual-only vs. multimodal, abstract vs. anthropomorphic displays). For each eHMI, we provided participants with an image and a short description of its functioning (see Annexes). This systematic selection aimed to guarantee that participants were exposed to the breadth of existing approaches at the time of the focus group, while keeping the total number manageable for group discussion.

### 4.3 Participants

We ensured that participants had a sufficient level of French to actively participate in the discussion, based on their self-assessment of their language level. No participants with disabilities were recruited, as no disabled person requested to participate.

The participants comprised six females and eight males, with an average age of 37 years old (minimum: 25 years old, maximum: 62 years old), and of different nationalities (nine French, two Belgian, four Luxembourgish and one Portuguese, including two persons with dual nationality). The majority of the participants lived in an urban environment (12 of the 14). Four participants had children under 10 years old, see Table 1.

**Table 1:** Profile of the participants to the focus groups.

**Group**	**Age**	**Gender**	**Nationality**	**French use (1-5)**	**Difficulty to walk**	**Children < 10 y.o**.	**Life envi-ronment**	**Car accident victim**	**Driving license**	**PBQ**	**PRQF**
1	29	M	FR	5	No	Yes	Urban	Yes	Yes	3.00	2.81
	53	M	FR	5	No	No	Urban	Yes	Yes	1.50	2.38
	37	M	FR	5	No	No	Urban	No	Yes	1.80	1.25
	36	F	PT	4	No	No	Urban	Yes	No	2.60	2.94
	35	F	LU	3	No	No	Urban	No	Yes	2.30	4.38
	32	M	FR	5	No	No	Urban	No	Yes	1.60	3.75
	62	M	LU	4	No	No	Urban	Yes	Yes	3.40	1.63
2	25	M	FR	5	No	No	Urban	No	Yes	2.40	1.69
	43	F	FR	5	No	Yes	Urban	No	No	3.00	2.88
	30	M	LU	5	No	No	Urban	Yes	Yes	1.90	5.25
	29	F	FR	5	No	No	Rural	Yes	Yes	2.50	5.94
	51	F	FR	5	No	No	Urban	Yes	Yes	1.50	2.00
	35	M	FR	5	No	Yes	Urban	No	Yes^*^	3.20	1.88
	31	F	BE	5	No	Yes	Rural	No	Yes	2.10	4.56

French use: self-evaluated from 5 (I use French all days) to 1 (I never speak French).

FR, French; PT, Portuguese; LU, Luxembourgish; BE, Belgian.

^*^ less than 3 years.

PBQ: Pedestrian Behavior Questionnaire (Deb et al., 2017b) measures the frequency of risky behaviors among pedestrians.

It consists of 20 items rated on a 6-point Likert scale (1 = very infrequently to 6 = very often). We present here the total average.

PRQF: Pedestrian Receptivity Questionnaire for FAVs (Deb et al., 2017a) assesses pedestrians' willingness to cross the road in front of a FAV.

It contains 16 items rated on a 7-point Likert scale (1 = strongly disagree, 7 = strongly agree). We present here the total average.

**Figure 4:**
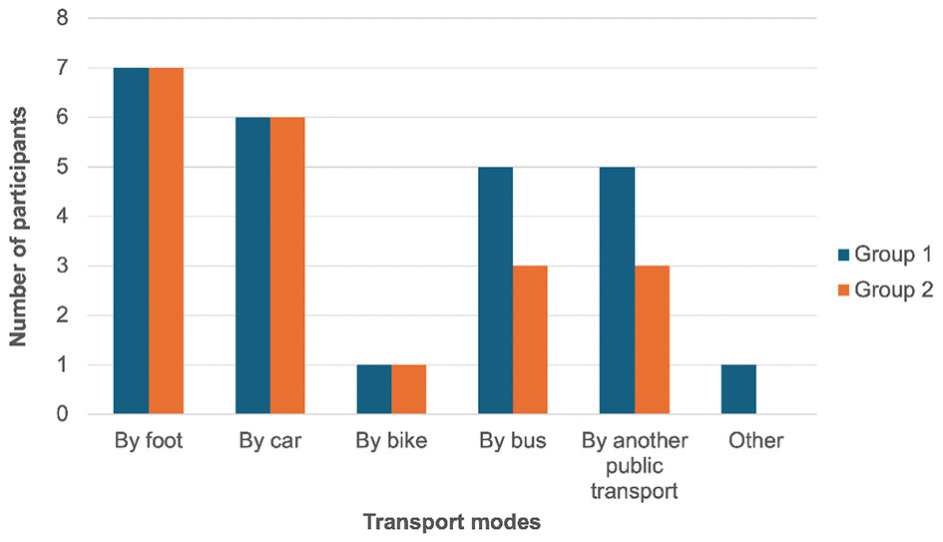
Daily transport modes used by the participants to the focus groups.

Concerning their daily journeys, they mainly walked (14), drove (12), and took the bus (8), but also rode a bike (2) and used other means of transportation (1), see Figure 4. The majority of the participants declared that they walked very often (11), while three often walked, two sometimes and none never walked. None of the participants had difficulty walking.

Half of the participants had already had a road accident as a driver or passenger. Most participants had a driving license (12).

The results of the global scores on the PBQ and PRQF questionnaires are reported in Table 1 and highlight a wide diversity of participant profiles, particularly with regard to their perception of autonomous vehicles.

### 4.4 Data analysis

After transcribing the exchanges and reviewing the notes written on the Miro boards, a thematic analysis was undertaken to select the main themes and group the comments by theme (Boyatzis, 1998). To achieve this, we followed the qualitative content analysis approach described by Schreier (2012). All data were carefully reviewed, and each segment was assigned to an appropriate category within a coding framework. The topic guide used during data collection served as a deductive basis for constructing the main dimensions, while most subcategories were generated inductively from the participants' contributions. The coding was carried out independently by two researchers, who then compared and discussed their schemes until consensus was reached, ensuring consistency and reliability in the categorization.

In the second focus group, after presenting each eHMI and asking participants to react and share their feelings and the limitations they identified for each eHMI, we collected the participants' votes for the most understandable and safest eHMIs, from their individual perspective. The votes were counted to ascertain the most understandable and safest eHMI.

In addition to the thematic analysis of focus group discussions, the responses to the PBQ and PRQF questionnaires were analyzed descriptively. For each participant, we calculated the global scores of both questionnaires, in order to provide an overview of pedestrian risk-taking behaviors and their receptivity toward FAVs. Given the exploratory and qualitative nature of this study, these results were not subjected to inferential statistical testing but were instead used to characterize the diversity of participant profiles and to contextualize the qualitative findings. The descriptive analyses were carried out using SPSS 27.

### 4.5 Ethical and legal issues

Ethical approval was not required for the studies involving humans because when the study was done (2021) the approval by an ethical committee was not mandatory in Luxembourg, and our institution internal ethical committee was created after this date. The studies were conducted in accordance with the local legislation and institutional requirements. The participants provided their written informed consent to participate in this study.

The purpose of the study and the use of results were explained to the participants. Then, they were informed that the results would be recorded and that they could stop or request that the withdrawal of their participation from the study at any time. Each participant was asked to electronically sign and send back the ethical and data management consent form before the beginning of the focus groups. All participants were over 18. At the end of the focus groups, participants received a voucher (50€).

The management of participant data in this study followed the framework established within the European research project in which it was conducted. This framework was based on a Data Management Plan and a Data Protection Handbook developed at the project level, in accordance with the General Data Protection Regulation (GDPR). In line with these principles, data collected during the focus groups were handled with care to preserve confidentiality. Participants were assigned pseudonymous identifiers, and only this pseudonymised information was used for research analysis. Access to the data was limited to the research team and storage was ensured on secure institutional servers. During the sessions, the collaborative platform Miro was used to support discussions and note-taking. No personal information was entered into Miro. Only participants' contributions related to the study topics were recorded. This ensured that the use of the tool did not compromise the confidentiality or security of participant data.

## 5 Results

This section presents the results of the focus groups. Results are based on Miro boards populated by participants and the discussions. The number of occurrences for each item is given in brackets for a total of fourteen participants. We counted them only when participants actively agreed, disagreed or commented on a statement. Verbatim comments were translated from French to English.

In the presentation of the results, the numbers in parentheses represent the number of occurrences for each topic, and p followed by a number indicates the speaker who emitted the verbatim used to illustrate the results. For instance, the participants check the *road signs and signals (12; “the presence of a traffic light reduces the risks, and it's safer”)* means that this point was raised by twelve participants and p3, on this subject said that “the presence of a traffic light reduces the risks, and it's safer.” And *they assess the presence of vehicle(s) (7)*, means that this item was mentioned by seven participants.

### 5.1 Current road-crossing behaviors

**Figure 5:**
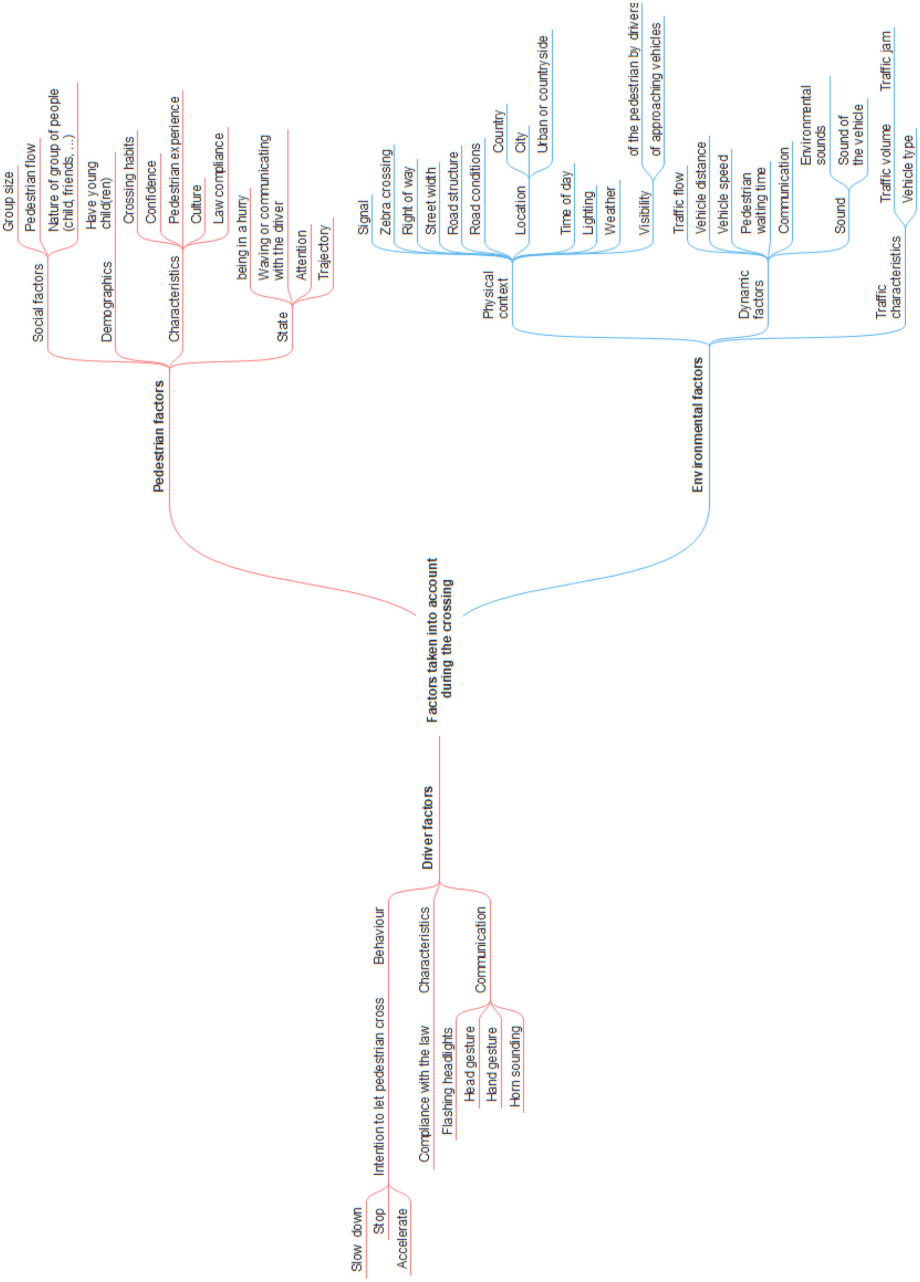
Factors considered by pedestrians when crossing; results issued from the first focus group. This image was manually produced.

The first focus group enabled us to identify the main factors considered by pedestrians in the current road-crossing context, see Figure 5.

Firstly, the participants check the **road signs and signals** for the following elements: firstly, whether or not there is a pedestrian crossing nearby (12: p3 “*the presence of a traffic light reduces the risks, and it's safer*”), secondly, whether there is a pedestrian traffic light and its color (3: p2 “*I have almost no control when the [pedestrian] light is green*”; p10 “*I'm not going to wait until [the pedestrian traffic light] is green*”), and finally, whether there is a button on the traffic light to ask for right of way or whether someone else has already pressed the button. The participants also drew attention to the fact that pedestrian crossings equipped with lights or LEDs are more visible to drivers and that pedestrians therefore feel more confident (4: p3 “*there are pedestrian crossings marked by LED lights on the pavement which make them easier to see for drivers and make us pedestrians a little more visible*”). The size of the pedestrian crossing (two or more lanes) also has an influence on their feeling of safety (2). In the case of a large pedestrian crossing, the presence of a pedestrian refuge island is reassuring (1). They also consider the time left to cross before the light turns red again and appreciate the traffic lights which give this indication (3: p5 “*in Brazil, there's a [pedestrian traffic] light with how many seconds left, so I check that too*”; p5 “*here the traffic lights take a very long time to turn green and you inevitably cross when it is red, I cross when it is red*”). Finally, they appreciate all signs and signals that encourage drivers to slow down, such as traffic lights that control speed and turn red if the speed is too high, speed cameras that indicate just when the speed limit is exceeded, speed bumps and intelligent speed bumps (4: p10 “*I feel more confident if the pedestrian crossing is raised, because the car is more likely to slow down*”). Ignorance of traffic signs by pedestrians is considered disabling (1: p12 “*I don't have a [driving] license and so I don't have the theory test, [...] and the fact that I don't understand certain signs, certain priorities, makes it difficult for me in certain areas when there are no pedestrian crossing signs*”). However, the presence of a pedestrian crossing, even with a green light, is no guarantee that pedestrians would cross, since they also consider other factors (3: p14 “*even if [the pedestrian light] is green, I wait for the car to stop before crossing*”).

When there is no pedestrian crossing, the participants say that they look before crossing (1), wait for a gesture from the driver (hand gesture or headlight flashing) indicating that they can cross (4 : p4 “*a gesture from the driver telling me that they are slowing down and that we can pass: a gesture of the hand or a flash of the headlights*”; p10 “*if there is a wave of the hand I will cross more confidently because I know that we have seen each other*”), or use the surrounding sounds to detect a possible danger (an oncoming car or an emergency vehicle such as an ambulance).

The participants stated that they looked both ways before and during crossing to **check the traffic and its intensity**. This means that they assess the presence of vehicle(s) (7), the density of traffic (10: p6 “*on a 50km road, if I see a lot of cars coming, even if I have the right to cross because there's a pedestrian crossing, I'm afraid to go*”; p12 “*I'll look at the traffic and cross even [if the light is red]*”), the speed of vehicles (6: p3 “*even when the [pedestrian] light is green, I still pay attention to the speed of oncoming vehicles if there are any, but there's less control*”), and the distance of vehicles coming toward them (2). This helps them decide whether they have time to cross. It should be noted that traffic density is not analyzed in the same way for each environment, i.e. in an urban or rural environment (3). Furthermore, when crossing a road with more than two lanes, drivers' reactions are better anticipated if traffic density is low (1). Noises and sounds are very important for detecting whether a vehicle is approaching and even whether it is decelerating or accelerating. Depending on how noisy a vehicle is, the pedestrian can detect the type of vehicle (3) and the driver's behavior (3). Sound is used more in backstreets (3: p8 “*for example, in my village there's a pedestrian crossing that's badly located, and it's mainly the noise that tells you whether it's safe to cross*”; p3 “*especially when a pedestrian crossing comes after a bend, you can tell by the sound whether a car is coming or not*”), whereas sight is mainly used on main roads. A noisier environment requires the pedestrian to be more vigilant (1: p11 “*If there's a polluting noise around, like a drill or a jackhammer, which doesn't let me hear the cars coming, that's one of the times when I'm more vigilant*”). At pedestrian crossings where visibility is reduced, sound can signal the approach of a vehicle. Participants raised the issue of electric cars, which do not make much noise and are difficult to perceive, particularly in car parks (6: p1 “*a few years ago I was almost run over by an electric car because I couldn't hear it*”). They were also concerned about the fact that FAVs will essentially be electric vehicles. However, some participants pointed out that they are used to having a lot of electric vehicles in their environment and that they now rely more on sight than on sound. Or, as electric car drivers, they are familiar with this sound and identify it more easily (2: p9 “*I drive a hybrid car, so I'm used to the noise of my car even though it doesn't make much noise*”). Another participant mentioned wearing headphones with ambient noise reduction to listen to music, which makes them pay less attention to sounds and more attention to sight (1: p11 “*I have headphones that reduce outside noise, so I don't pay attention to noise anymore*”).

The **context and environment** are also taken into account (2: p5 “*roadworks and the state of the road also have implications*”), for example, whether the road in question is a small alley, a main road or a low-traffic area where pedestrians have priority (called “*zones de rencontre*” in French, vehicles are allowed to travel in these zones, but at a reduced speed (20 km/h), and pedestrians are allowed to walk on the road. They have priority over vehicles, but must avoid obstructing traffic). They will cross more spontaneously in an alleyway because they will always have time to run across if a vehicle is coming (6: p5 “*if there is a car, we have time to react fairly quickly because we can walk faster*”; p13 “*on a road with four lanes I will look for a pedestrian crossing more than in a small street*”). The participants agreed that they are more cautious in large towns than in small villages (3: p12 “*in a large street where there are several lanes and a lot of traffic, we will be more careful than in a small village in the countryside or in small streets with good visibility*”). But they noted that there are major differences between habits in certain towns (for example, it seems safer to cross in Brest (France) than in Paris (France) – 5: p12 “*in a large street where there are several lanes and a lot of traffic, we will be more careful than in a small village in the countryside or in small streets with good visibility*”), and even between countries (for example, it seems safer to cross in Luxembourg than in France – 5: p13 “*in France people pay less attention if there is a pedestrian crossing while in Luxembourg as a general rule people stop if there is someone at the pedestrian crossing and they want to cross*”; p10 “*if I am abroad in a completely different culture I would not take the risk of crossing if there is no pedestrian crossing*”). The visibility of the place where the pedestrian is crossing is also important, i.e. if there are parked cars, street furniture or buildings blocking the pedestrian's view, or if the pedestrian crossing is not easily visible for vehicles (3: p8 “*if it's a place where I don't have much visibility, I prefer to go further*”). Weather conditions are also a key factor. If it is raining or snowing, participants say they are more cautious because they could slip, or the vehicle could have more difficulty braking (4: p10 “*I pay attention to the weather, because if it snows or if there is rain, I know that the vehicle will have more difficulty braking and that I too would have more difficulty crossing at a pedestrian crossing if I had to hurry*”). Opinions on crossing at night are divided (6: p8 “*I have a black coat, and I'm more afraid that vehicles won't see me, so I'm more vigilant*”; p11 “*cars have headlights so we will cross more easily if we see that there is no car; on the other hand we are seen much less, and therefore it is more dangerous*”; p10 “*I have more difficulty crossing at night because I rely a lot on the vehicle approaching, seeing the state of mind the driver is in and at night I am unable to get this information*”). Some participants thought they were safer because vehicles could be seen better thanks to the headlights. But above all, participants do not trust themselves at night and therefore rely less on their senses. They cannot see the drivers and predict their attitudes, and they are afraid of not being seen, for example, because they are wearing a dark coat. They therefore try to find a lamppost in order to cross in the light and be seen better (1: p12 “*I'm going to try to cross a street with a lamppost*”).

**Being familiar with the area** reassures people and puts them at ease, because the usual traffic patterns and driving habits in certain streets are known (2: p9 “*when it's a familiar place we know the traffic, if it's faster, denser traffic [...] we can anticipate the traffic on that street*”). However, in this case, there is a risk of relying more on habits and less on senses (1: p10 “*the street right next to my home that I know very well and which has very little traffic, I sometimes cross without looking at all because I know that normally there is no one there but once or twice, I was surprised because a car was coming*”). For example, in little-frequented alleyways, some participants cross without looking and have already been caught out.

**Driver behaviors** are also observed. The participants noted that they can easily identify drivers who intend to let them cross by checking whether they are using their telephone (2: p11 “*if he's on the phone, I'm not going to take that risk*”) or focusing on the road (2: p9 “*the person in the car and their attention*”), and observing the driver's attention and their behavior (6), including whether they show any sign of slowing down or stopping. They have already observed that some drivers speed up to make it clear that they have no intention of stopping (2: p8 “*we immediately see the people who intend to stop or not: there are those who ignore us who act as if they had not seen us, there are those who see us and who all of a sudden start to slow down and there are even those who accelerate*”). As pedestrians, they check whether the driver has seen them, and generally wait for vehicles to come to a halt or almost to a halt before stepping into the road. They also note that the driver's attitude takes precedence over the type of vehicle (4: p13 “*it can be a mistake to base it just on the engine capacity because I have already seen people who drive in minivans with children in them who were less careful than people who drive with big BMWs or Mercedes, for me, it's not a criterion of vigilance*”; p11 “*I'm going to pay attention to the driver and his attitude*”).

The **presence of other pedestrians** has an impact on their behavior. The participants noted the existence of a group effect (6: p4 “*for example in Paris, the whole group will go, even if the light turns red in the meantime, all the pedestrians will go across and block the drivers, until all the pedestrians have passed*”; p5 “*I feel more protected when there is a large group, because it is more visible to the driver*”). This means that if other pedestrians are waiting, they often wait too. On the other hand, if other pedestrians cross, this influences them and encourages them to cross. To explain this, they argue that a group of several pedestrians is better seen from a distance, which is reassuring (2: p11 “*it's a larger mass and more visible to the driver, so it's reassuring to have a group movement*”). The presence of friends will encourage them to wait longer to cross, to be sure of crossing safely together and not being separated (1: p13 “*if there are several of us, and we decide to cross by ourselves, and we are with friends, and they do not cross, we feel a little stupid with each waiting on one side of the road, so by default we would rather wait*”). In addition, there is a balance of power between vehicles and pedestrians. This means that if there are a lot of vehicles, pedestrians tend to stand back and look for protected crossings. On the other hand, if there are a lot of pedestrians, if a few of them decide to cross, the others tend to follow, because they think that the driver will feel obliged to stop for a group. It is therefore seen as an opportunity. The presence of a young child (accompanying the pedestrian) makes them more attentive and causes stress because the child may not be seen by the drivers (3: p9 “*if I'm with my son, it has to be a little educational, so no I don't do that [crossing on red], but if I'm alone, I do like [the others, I cross on red]*.” Thus, they avoid crossing outside pedestrian crossings and hold the child firmly by the hand or carry the child to show that they are accompanying children (p9 “*my child [(5 years old)] is smaller than the cars and is less in the drivers' field of vision and if there is a lot of traffic, I will carry him so he can be seen by the cars*”).

While the **type of vehicle** (bus, lorry, bicycle, type of car, etc.) makes a difference for the participants, they did not agree on which type of vehicle they considered the most dangerous (3: p10 “*if I see a big sedan coming that's going fast with a rather young driver, I know I have to be a little more careful*”).

Some participants indicated that **they signaled** (e.g., by waving) to the driver to indicate that they were going to cross the road (5: p6 “*I make a gesture to make him slow down and when I cross, I also make a gesture to thank him*”).

Factors other than those mentioned above are considered when **crossing the road away from a pedestrian crossing**. Being in a hurry encourages people to cross any way they can (1: p13 “*it depends on the mood*"; p13 “*if I'm a little in a hurry, I'll [cross on red] if I'm a little relaxed, I'll wait for the light*”). In this case, the participants admit that they give priority to finding the shortest route. A pedestrian crossing too far away is also seen as a good reason for crossing away from a pedestrian crossing (1: p1 “*If it's not too far, if I have to walk 500m to the pedestrian crossing I don't take it*”).

### 5.2 How do you imagine a future with FAVs?

**Figure 6:**
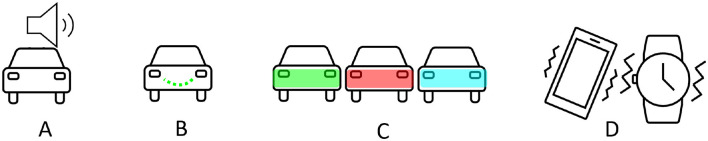
Participants' ideas raised during the first focus group about communication methods between the FAV and pedestrians. **(A)** Sound signal. **(B)** the FAV “smiles” to indicate that it has recognized the pedestrian and is letting them cross. **(C)** LEDs illuminating the FAV, with each color having a meaning (e.g., you can cross, I will not stop, etc.). **(D)** Vibrating devices indicating that it is safe to cross the road.

Before introducing the eHMIs, participants stated that a **communication method is needed between the pedestrians and the FAVs**. For this, participants suggested adding sound (3: p5 “*beeps, something audible that assures us that the [autonomous vehicle] has seen us and will stop*”) or LEDs that form a smile (1: p12 “*little LEDs with a little smile to humanize the car*") or change color (4: p5 “*the LEDs change color to show that it has detected nearby pedestrians*”), or using flashing headlights (2), or a device, like a smartphone or a smartwatch, that vibrates to indicate when it is safe to cross the road (1: p13 “*as someone who wears a connected watch or a phone, arriving at a pedestrian crossing and when I can cross my phone receives a notification or my watch emits a small vibration that tells me you can go ahead, you've been spotted, that would give me confidence*”), see Figure 6. Participants suggested a combination of several sounds or visual signals to cater to people with disabilities (1: p11 “*that appeals to many senses, obviously I'm thinking of people with disabilities too*”). However, they noted that, even today, many people do still not use smartphones or connected watches, or do not know how to use them; this is known as the digital divide (1: p11 “*I'm thinking of all the people who are not connected and are computer illiterate, who don't have a smartphone, and we need to find other solutions*”).

**Figure 7:**
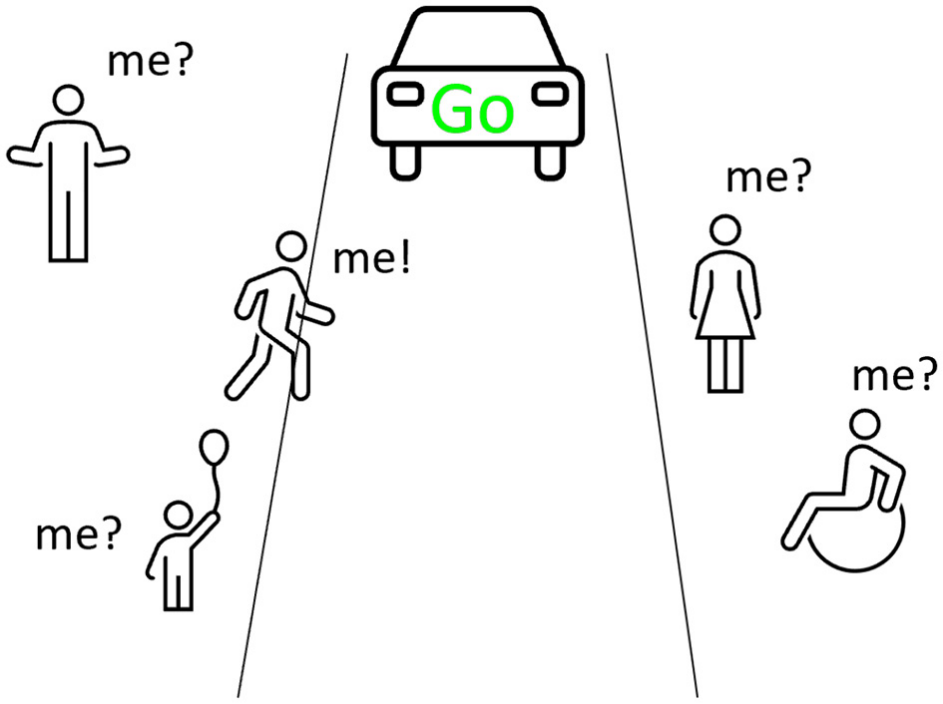
Problem identified by the participants during the first focus group: for whom is the signal emitted by the FAV intended if several pedestrians are in position to cross the road in front of the FAV?

However, the following question still remains: when several pedestrians are on the pavement, and the FAV sends a signal, how will they identify who the signal is intended for (see Figure 7) (1: p1 “*I'm not the only one on the road, there are several of us pedestrians, how do you expect the autonomous car to tell me and all the other pedestrians that it's passing?*")?

The participants also imagine that communication between FAVs and pedestrians will be necessary when FAVs are first introduced into traffic, but that it will become obsolete after a certain time because pedestrians will have learned how to interact with FAVs and will have developed their confidence (1: p5 “*I think that in the beginning it's important to develop trust between the autonomous car and people. After 5 or 6 years, people will trust it so much that they won't need visual feedback anymore*”).

**Figure 8:**
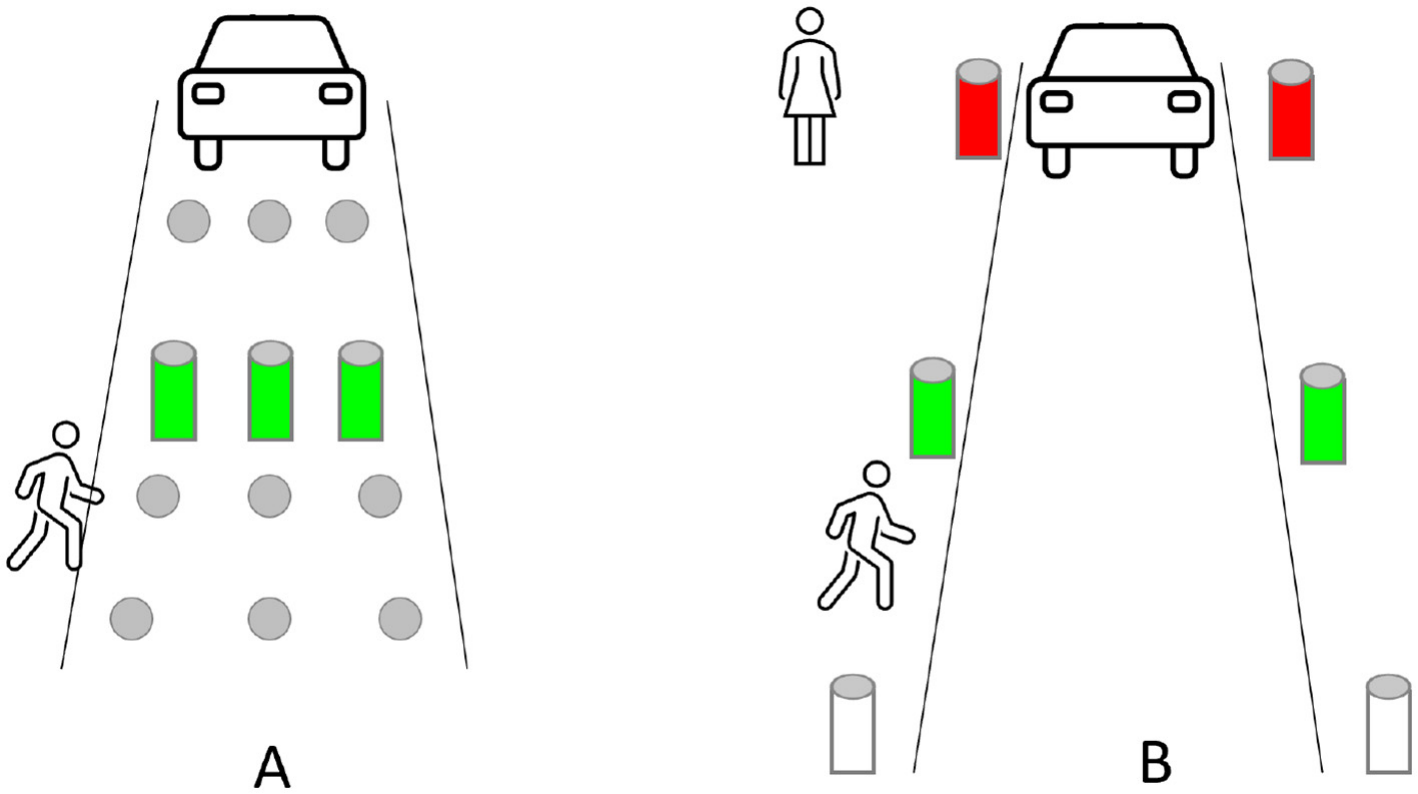
Participants' ideas raised during the first focus group about the use of the infrastructure. **(A)** Some bollards rise up, indicating that the pedestrian can cross safely. **(B)** Some bollards change color, indicating whether it is safe (green) or unsafe (red) to cross the road in front of the approaching FAV.

Participants also proposed that the communication appears on **indicators on the street** (3: p10 “*I wouldn't like it to be the car that warns me that I can cross, but rather an indication at the pedestrian crossing that tells me that the cars are aware that I'm coming and that I can cross*”). For instance, illuminated bollards could indicate to pedestrians when it is safe to cross, see Figure 8B. Or some bollards could rise up to provide a safe path and show that the FAV will stop (1: p13 “*if you press the light, the car knows it and a brick goes up to block the pedestrian crossing and as a pedestrian you know you can cross*”), see Figure 8A.

Participants also said that they expect **technology to be humanized and designed from an emotional point of view** (1: p12 “*to replace the driver, put emotional design onto the car*"), and that the FAVs should take into account that a pedestrian is first and foremost a human being with unexpected behavior.

Lastly, participants imagined that FAVs **would respect the Highway Code better than human drivers**, particularly in terms of speed (1: p3 “*if there are more autonomous cars, they are more likely to respect speed limits*") and giving way to pedestrians (slower vehicles and better anticipation) (3: p3 “*having been next to autonomous cars in operation, they will anticipate strongly and slow down as soon as there is a dangerous situation*"). Their large numbers should make the roads safer.

**Figure 9:**
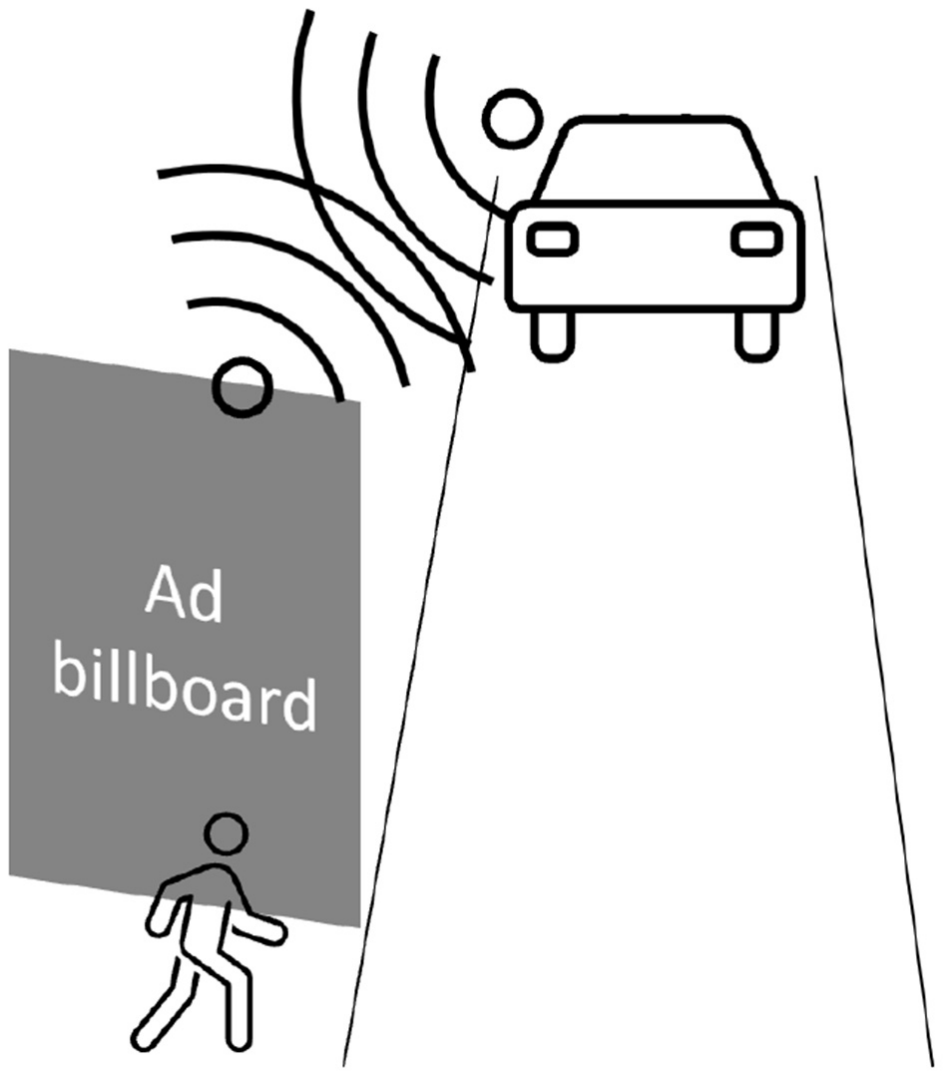
Participants' idea during the first focus group: communication between billboards and FAVs can help detect pedestrians obstructed from the FAV's view.

The pooling of sensors from FAVs and indicators on the street should make it possible to **detect all pedestrians, including those obstructed from view** by billboards and bus stops (1: p3 “*I find it interesting that street furniture can inform autonomous cars about the environment, for example to see behind a pole [...] to network street furniture and vehicles*"), for example, see Figure 9.

**Figure 10:**
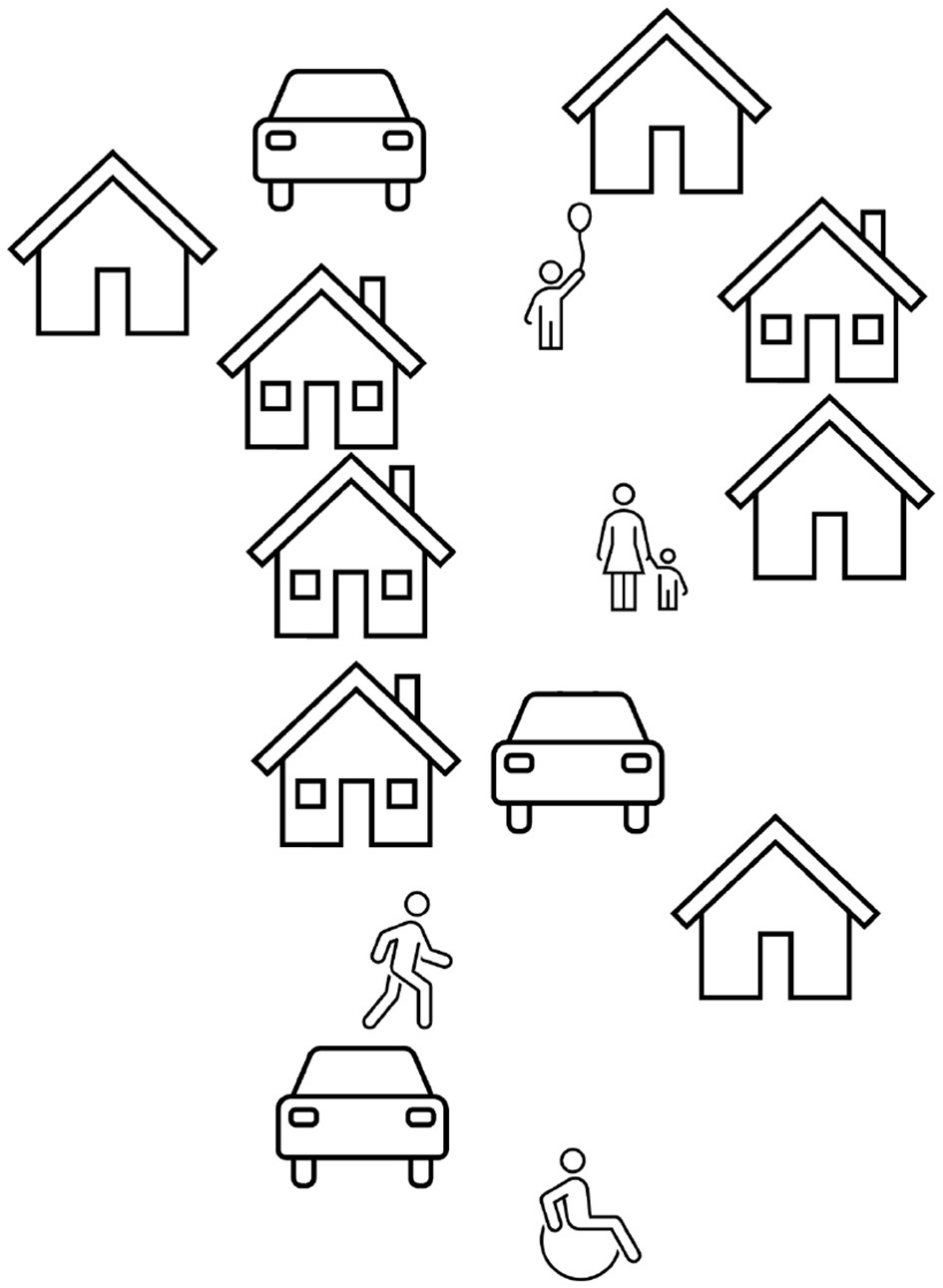
Participants' idea during the first focus group: no more separation between the road and the pavement. Pedestrians walk wherever they want, and the FAVs detect them and stop whenever necessary.

As pedestrians should have priority in any situation, focus group participants believed that the urban landscape would evolve into a **low-traffic areas** (shared spaces or “*zones de rencontre*” in French) and pavements may no longer be necessary (1: p1 “*I wonder whether 30 years from now there will still be a need for pedestrian crossings because cars have to recognize pedestrians. I see a large area where everyone can walk and drive, and the pedestrians remain the priority, with no pavement*"), see Figure 10. They even asked if it was necessary for the car to communicate with pedestrians, since it could be interesting if it was instead up to pedestrians to give the signal to the FAV that they were going to cross (1: p6 “*It's up to the pedestrian to signal that he/she wants to cross, it's like the gesture we used to make to the driver*").

### 5.3 Acceptance of FAVs

#### 5.3.1 Interest of FAVs

**Figure 11:**
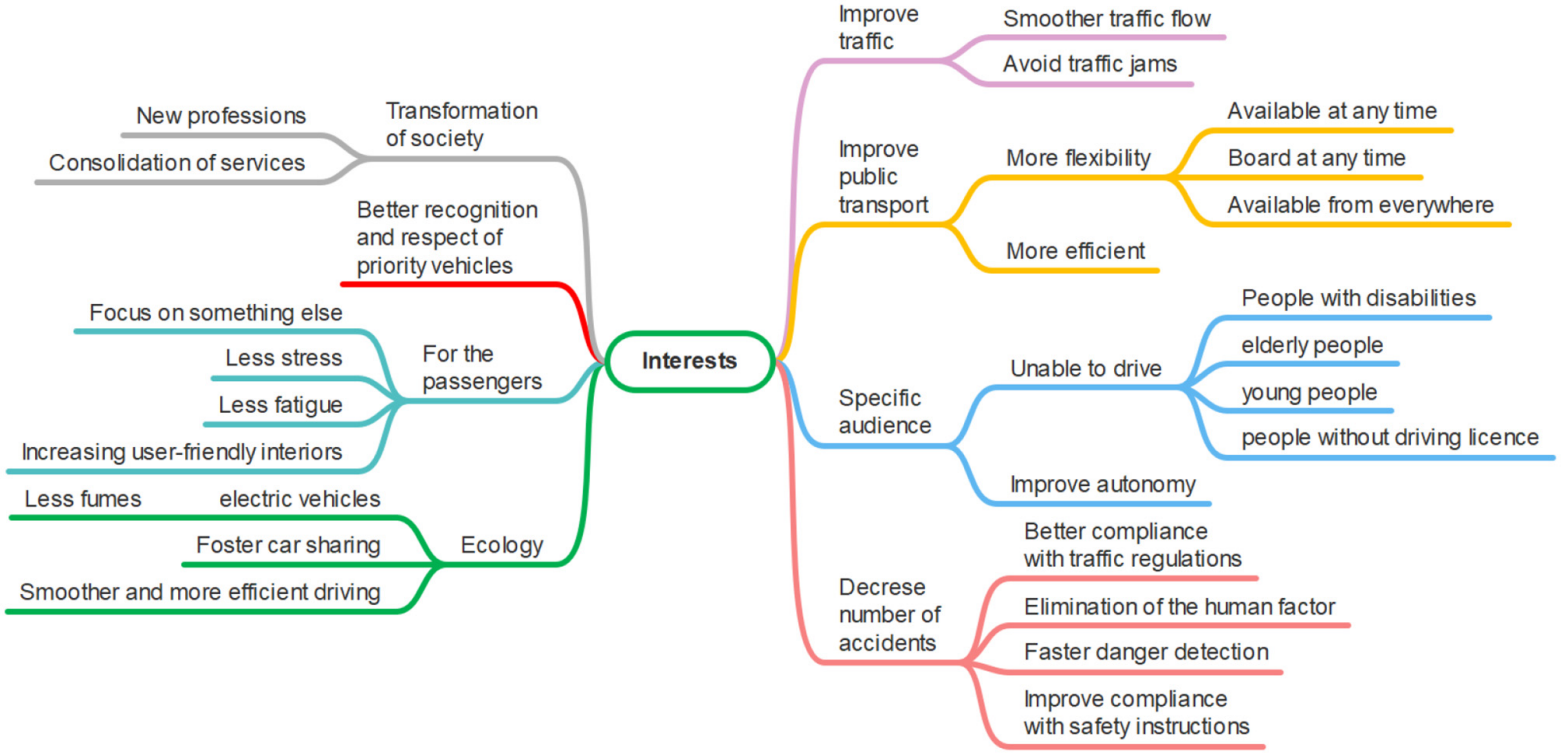
Interest of FAVs, first focus group results. This image was manually produced.

The participants noted the following arguments in favor of FAVs, as also shown in Figure 11. They believe that FAVs could **smooth the traffic flow and reduce traffic jams**, in particular, thanks to a generalization or facilitation of **car sharing** (2). This could also have an impact on the **reduction of transport costs** (1: p5 “*there could be fares for sharing autonomous vehicles, which would be cheaper*").

Participants have certain expectations of **public transport using FAVs** (4). These include the possibility of boarding the vehicle at any time, due to there no longer being specific bus stops, and the possibility of finding a means of transport (like a taxi) to the desired location at any time. Therefore, they mainly expect public transport to be flexible (timetables, stops, destination), while offering the possibility to easily connect destinations not currently served because they are too isolated (p2 “*have more efficient public transport*"; p3 “*review the concept of buses with fixed timetables, have flexible transport on demand*"; p5 “*give more possibilities because in certain places where there is one bus per hour, people give priority to the car whereas if we have more flexibility and offers we might move toward a shared vehicle*"). If these expectations are met, it will lead to a more efficient public transport system and, therefore, more widespread use.

FAVs could also take in consideration **specific audiences** (1), like people unable to drive current vehicles, including those with disabilities, elderly or young people and people without a license (p3 “*new services, such as transport for elderly people who can't necessarily drive*"). FAVs will facilitate their travel and increase their autonomy (p1 “*I see it as an aid, to get to the shop or the park, so you have contact with everything*").

Participants also think that FAVs could **reduce the number of accidents** (2) thanks to better compliance with the traffic regulations compared to human drivers (e.g., respecting speed limits, stopping at a pedestrian crossing), the elimination of the human factor that causes some accidents (alcohol, fatigue, drugs, lack of concentration particularly due to smartphones, panic and lack of vehicle control, etc.), a faster danger detection, and decreasing non-compliance with safety instructions (like entering a bus even when the doors are closing), as it is not possible to negotiate with a machine (p7 “*technology will make fewer mistakes than humans"* ).

In addition, the **ecological factor** was mentioned (3), as FAVs are expected to be electric cars and there will therefore be fewer exhaust fumes (p3 “*an autonomous car will probably be electric and therefore produce fewer exhaust fumes*"). Furthermore, transportation will have a lower impact on our carbon footprint, due to the increase of car-sharing among the population, and the smoother and more efficient driving experience, thanks to the more ecological driving style of FAVs (p5 “*more people will take a car, not alone but shared with someone else, which may also reduce pollution*").

For **passengers** (1), the positive consequences could be the ability to focus on something other than driving, less stress, and less fatigue on long journeys (p2 “*I can leave at any time, even if I'm drunk*").

Some participants expect that FAVs will be able to **better anticipate the arrival of emergency vehicles**, such as ambulances, police cars or fire engines, and let them cross more quickly.

In conclusion, participants expect a **radical transformation of transport and society** (2) with the emergence of new professions, consolidation of services and increase of user-friendliness in cars with reinvented interiors (p2 “*the driver's role [in public transportation] is being transformed into a job that encourages collaboration in the vehicle, that can help people to get in or provide a service other than having a steering wheel in their hands, meaning added value for passengers*"; p3 “*at the beginning, a lot of teaching will be needed, and so people will have to be accompanied to explain how the service works, and helpers will be needed for people with reduced mobility*"). FAVs will also bring the question of car ownership to the forefront, by pushing toward car-sharing.

#### 5.3.2 Risks or problems of FAVs

**Figure 12:**
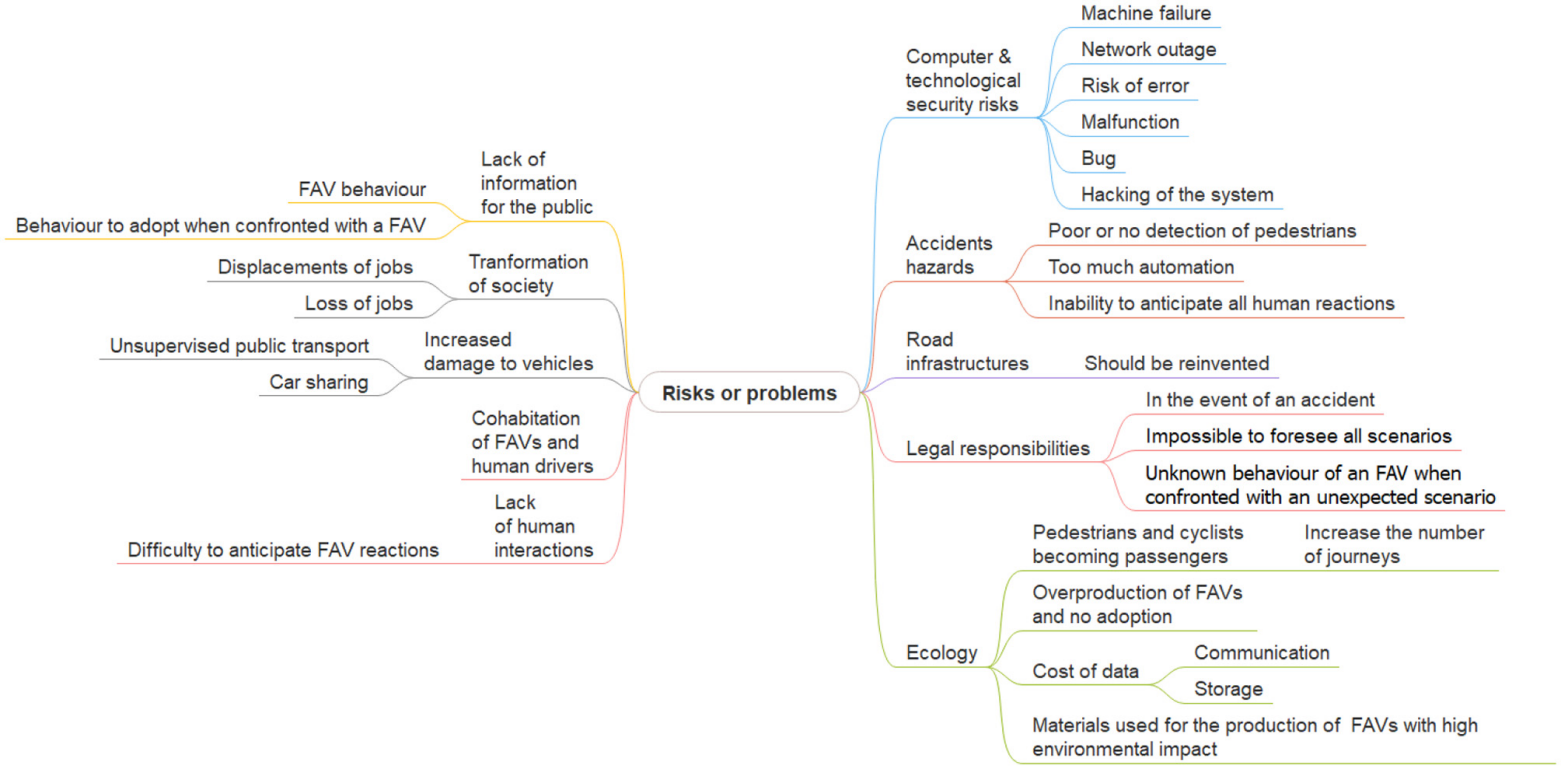
Risks and problems with FAVs identified during the first focus group. This image was manually produced.

Although participants see many positive reasons for deploying FAVs, they also mention risks and problems, see Figure 12.

Several **computer and technological security risks** were raised, such as machine failure, network outage, risk of error, malfunction of the control system, bugs or algorithm errors, or even the hacking of the control system.

Participants think that some serious **accident hazards** exist, like no detection or a poor detection of pedestrians, FAVs abandoned in random locations after use, which could be dangerous for other vehicles (2: p5 “*there should be terminals to regulate where autonomous cars are left, not like electric scooters which are abandoned anywhere*”; p3 “*like hire cars in Paris with dedicated parking spaces*”; p3 “*[the autonomous car] could join a dedicated car park*”) or too much automation, creating additional accidents [e.g., the automatic closing of doors in a bus was cited (4): p1 “*in an autonomous taxi I've already got stuck in a door*"; p5 “*it will be like the underground with a sound to indicate that the doors are going to close*"; p3 “*the passenger has to give a lot of feedback to the car in my experience*"]. Participants question whether the system will always be able to anticipate human reactions, particularly pedestrians' reactions, and thereby avoid an accident, i.e. a child playing with a ball who suddenly throws it into the road.

Concerning **road infrastructures**, there is a consensus that they need to be reinvented to be suitable for this new paradigm.

The **legal responsibility** in the event of an accident was a further issue raised by participants. This must be defined with regard to ethics. Participants ask who is responsible in the event of an accident, and how the vehicle decides between two scenarios if an accident is inevitable. They also think that it is impossible to foresee all possible scenarios, and in this case, question how the FAV will react to an unexpected scenario?

Concerning **ecology**, participants note that pedestrians or cyclists may become passengers because using an FAV will become more comfortable and better adapted to their needs than using a car currently (2: p3 “*Won't people who don't currently have a car use this form of transport more, whereas before they used to get around more on foot or by bike?*"; p6 “*Overuse of the autonomous car over short distances (5–10 km) as a result of a fashion effect, a mass effect?*"). This will increase the number of journeys. They are also afraid that FAVs will be only a fad, meaning that many FAVs will be produced at the beginning, thinking that the market will follow, but will then be put into storage due to lack of use (1: p3 “*like electric scooters, the risk is that it will become fashionable to use autonomous cars and everyone will want them, then the market will collapse and there will be a lot of autonomous vehicles that will have been produced [not to be used] and there will be an environmental cost*"). Furthermore, the cost of data used, their communication and their storage should be examined (1), as well as the material used to produce them (1: p3 “*the construction of an autonomous vehicle will have a greater ecological cost*").

For the participants, the **lack of human interaction** between the (missing) driver and pedestrians is a risk (1: p4 “*I have the impression that a highly automated society will isolate people who are already isolated. In the sense that if I can take an autonomous vehicle where I'm on my own, we won't talk to the driver, the delivery people, etc*.”). It will also make it difficult to anticipate vehicle reactions.

Participants also noted that the **cohabitation of FAVs and human-driven vehicles** will generate risks and accidents.

They think that there is a greater risk of **human-invoked damage** to equipment by unsupervised users in public transport and car-sharing.

Participants believe that FAVs will change society by **displacing jobs** (and will therefore cause job losses).

From the participants' point of view, the population has **not been sufficiently informed** about FAVs, particularly the behaviors of FAVs and the behavior pedestrians should adopt when confronted with an FAV.

#### 5.3.3 What would allow FAVs to be seen as reliable vehicles?

**Figure 13:**
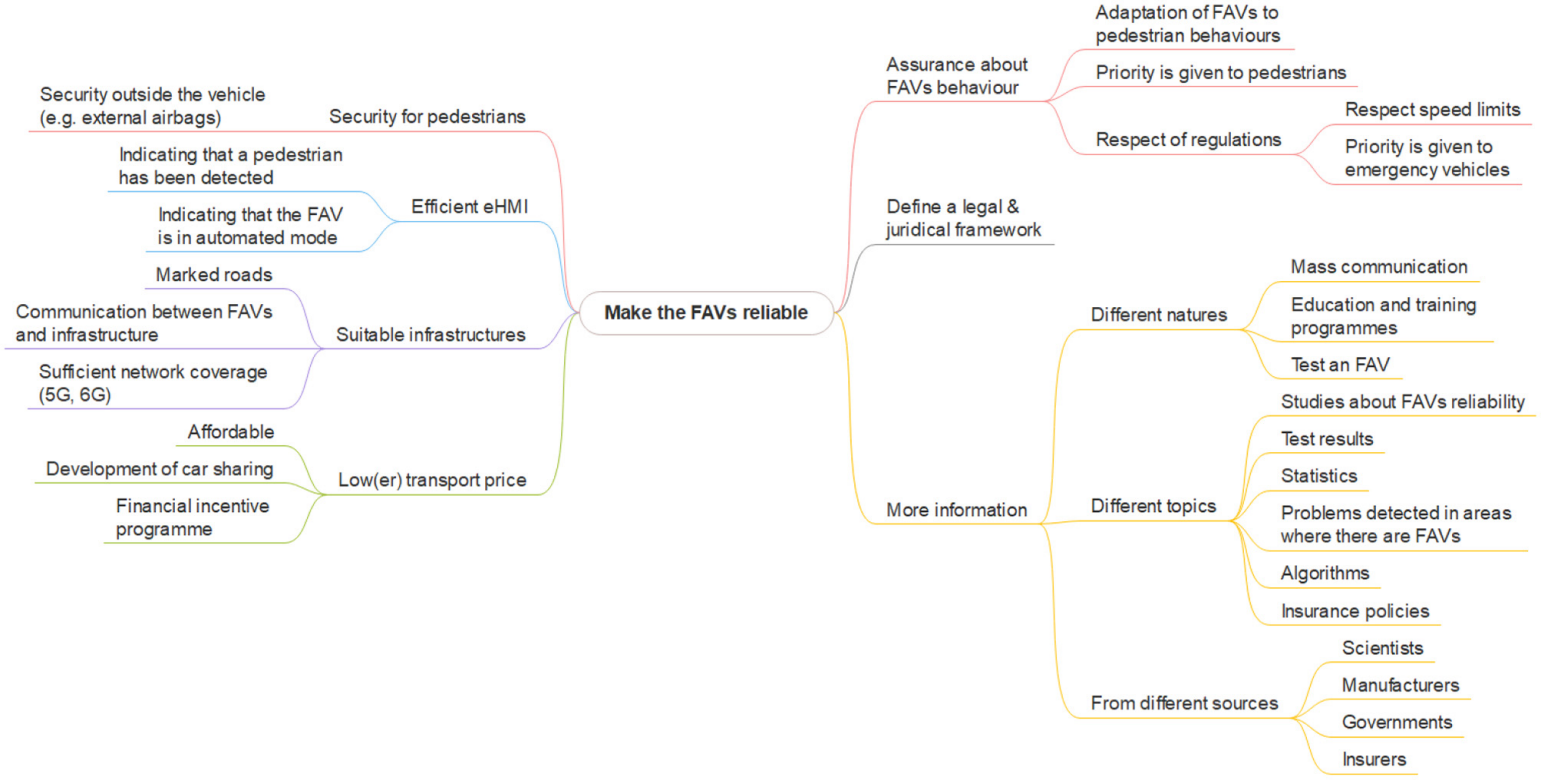
Factors contributing to the reliability of FAVs, first focus group results. This image was manually produced.

During the first focus group, the participants identified several elements that could have an impact on their acceptance of FAVs, see Figure 13.

The participants reported a **lack of information** about FAVs (5). They call for manufacturer and scientific studies demonstrating the reliability of the systems, test results, reliable statistics including accident rates with and without FAVs, and feedback on the problems detected in the areas where FAVs are introduced in the form of a mass communication campaign to all inhabitants of the areas affected by their introduction (p6 “*have they tested, have they checked, what have they planned, which areas or regions will be explicitly affected*"; p7 “*I'd like to know if there will be fewer accidents*").

Participants also think that **algorithms should be made public**, in order to be transparent about how the system works and how it makes decisions, and in particular, to know what will happen if an FAV cannot brake to avoid a pedestrian (3: p4 “*we could make the vehicle's behavior public: how it's supposed to work, what happens if a pedestrian crosses the road, how the vehicle is supposed to break, what the vehicle's behavior is*"; p4 “*will the pedestrian or the passenger be favored in the event of an accident*"; p3 “*what is the decision-making process in the event of an accident*"; p2 “*the transparency of the algorithm*").

People also need to be assured about the **behavior of FAVs**. They expect that when an FAV detects a pedestrian wishing to cross the road, the piloting system will automatically stop the vehicle, and the FAV will behave in compliance with the road regulations. Furthermore, they expect FAVs to be able to operate in dangerous areas (heavy pedestrian traffic, areas where pedestrians do not respect road regulations, etc.).

The **legal and juridical framework** must be defined (1: p3 “*if there's a legal framework, it means that society as a whole has thought about the concept of the autonomous car and that a lot of things have been anticipated, and I'll feel safer in the sense that if we know who's going to be responsible in an accident, it means that we've thought about what accidents are going to happen in what conditions, what we're going to do, and so on. So, we've thought things through and tested the limits of different vehicles. And perhaps as a result, there are only vehicles on the market that are relatively safe because there is a legal framework governing that*").

Furthermore, as **insurers** will have to bear the financial consequences of accidents involving FAVs, if they engage with this subject and demonstrate a position of trust toward FAVs (e.g., agree to insure FAVs, lower insurance premiums, etc.) then the participants would be more confident (1: p2 “*insurers are the ones who bear the financial consequences of accidents; when insurers get involved in the subject, it will be a mark of the reliability of autonomous vehicle operation*").

To build confidence in FAVs and become familiar with them, participants suggested several **education and training** programmes, including the safety measures and behaviors to adopt when an FAV approaches them (2: p3 “*show and explain the safety measures, communicate and teach people not to be afraid,..., this work needs to be done, because it's a new means of transport*"; p6 “*a reminder of the correct behavior to adopt in order to comply with a safety-related regulation*") and the opportunity to try FAVs free of charge. The governments of states where FAVs are in circulation must communicate officially about FAVs to increase confidence among the general public.

The participants also raised **financial** questions. The FAVs will be better accepted if they are affordable, if the possibility of sharing an FAV reduces the price and if employers push for their use, for instance via mobility bonuses (1: p3 “*if there are private [employers] who encourage employees to use [autonomous cars] in the same way as they can encourage them to use public transport and bicycles*").

It was also noted that providing a **suitable infrastructure for FAVs** and all other road users in an environment with FAVs is a key factor in their adoption. For this, all roads must be marked with a common standard. Communication between FAVs and infrastructure items like traffic lights must be ensured, as must good 5G or 6G coverage in areas in which FAVs are authorized, including tunnels or underground car parks.

Finally, the participants raised the question of **communication between FAVs and pedestrians**, for instance, indicating that the pedestrian has been detected by the FAV, or that the vehicle is in automated mode.

The **security of pedestrians** must be ensured, for example, by incorporating external airbags: p6 “*the safety that is currently inside the vehicle must be transposed to the outside for pedestrians, for example external airbags*.”

### 5.4 eHMI preferences

During the second focus group, the participants agreed that FAVs should **communicate with pedestrians**. According to their discussions, communication channels between the FAV and pedestrians should be multiplied to ensure that everyone can understand the message (including children and those who are visually impaired, color-blind, illiterate, unfamiliar with technology or do not have a smartphone, “*It seems that all the solutions are designed for standardized people and do not take into account people with disabilities. How will people who are drunk, or drug addicts understand all these situations?*,” about a textual eHMI: “*The text interface is problematic for children and the visually impaired*.”) using, for instance, visuals and sounds. Participants pointed out that text should be avoided or supplemented with other information, such as icons (for children, and those who are illiterate or do not read the language of the country and who will therefore not be able to read the text, “*Useful drawing for people who cannot read or understand the language (including children) and sounds or vibration (for blind people*,” “*the cane should vibrate*”). They also mentioned that, if there is text, it should be short to allow it to be read quickly. Participants stressed the importance of using current and well-known means of communication wherever possible, and not to repeat the information by any other means. For instance, they referred to existing conventions, such as the indicator to show that the vehicle is going to turn, white tail lights to indicate that the car is going to reverse, etc. Finally, participants emphasized that communication by a light signal must consider the needs of color-blind people, i.e., a change in color of the message must be accompanied by a change in position or shape, as for traffic lights (about a LED eHMI: “*For visually impaired and color-blind people, the ‘go' and ‘stop' signals are positioned in the same place, which poses a problem*”).

**Figure 14:**
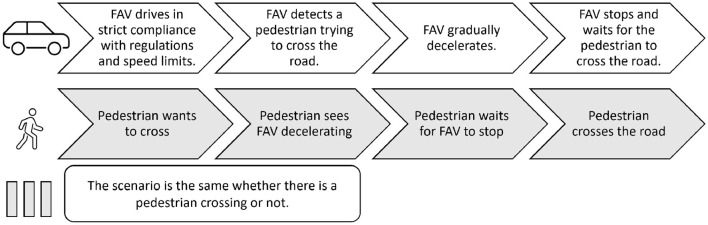
Behavior of the FAV and pedestrian in a crossing situation envisioned by the participants during the second focus group.

From the point of view of the focus group participants, the **behavior** of the FAV should respect the following propositions, see Figure 14: (1) the FAV should always let pedestrians cross, even without a pedestrian crossing. (2) In this case, it is only necessary to communicate with pedestrians in extreme cases (when the vehicle cannot stop). (3) The FAV must slow down progressively to show that it has detected the pedestrians and will let them cross. (4) It must be ensured that vehicles respect traffic regulations, including speed limits. (5) FAVs should stop far enough away from pedestrians to make them feel safe.

Current **infrastructure** features should be not only kept and favored (because they are well-known), but also improved. Traffic lights should detect the presence of pedestrians and turn green quickly for pedestrians to avoid waiting.

Finally, the participants **voted for the safest and the most comprehensive eHMIs** and also suggested improvements for them. The eHMI with projection on to the road is the one preferred by participants, from the points of view of both safety and comprehensibility. The results for the highest-scoring eHMIs for each category are available in the table below (see Table 2).

**Table 2:** Highest-scoring eHMIs for each category.

**eHMI**	**Compr**.	**Safe**	**Suggestions for improvement**
**Projection 1**†	14	14	- Ensure good visibility even in sunlight. - Connect canes for the visually impaired with the system so that they indicate when it is safe to cross.
**LEDs 1** see (Dey et al. 2018) Figure 2	10	10	- Make the warning light present all around the vehicle when it is red or green so that it can be seen from anywhere. - Make the warning light red if there is a danger but the vehicle will not stop, and red and flashing when the danger is too great, e.g. the vehicle will not stop and there is not enough space for the pedestrian to stop easily. - If a pedestrian is detected, ensure that the car stops and lets the pedestrian pass in all circumstances without displaying any information. - Keep the red signal only.
**Other 1**††	9	12	- Raise a barrier across the road. - Ensure that the barrier rises quickly. - Ensure that FAVs detect pedestrians crossing outside protected crossings.
**Icon 1** see(Clamann et al., 2017)	11	7	- Modernize the design of the display and the icons. - Do not display the speed of the vehicle. - Protect the display to prevent it from being damaged, e.g., by a stone. - Ensure that the display is in color.
**Textual 3**†††	8	8	- Display the message on all sides of the vehicle. - Do not use multiple colors in the same message. - Make the text concise.

*N* = 14. Compr.: total votes for more comprehensive. Safe: total votes for safety.

†: World premiere of the Mercedes-Benz F 015: Luxury in Motion at the CES, Mercedes-Benz Media, 2015 Mercedes F015

^††^: SAAQ, 2019 SAAQ

^†††^: The self-driving car you can't miss: Drive.ai, C. MacDonald, Daily mail, 2018 Drive-ai

## 6 Discussion

In this section, we first sum up the observations made during the study, then discuss them in comparison with other studies, where possible. We also identify the limitations of this study.

The observations are organized in a way that answers the research questions.

### 6.1 RQ1: What are the key factors considered by pedestrians when crossing the road today?

When crossing the road today in front of a human-driven vehicle, pedestrians take several key factors into account: factors relating to themselves, such as social factors, demographics, characteristics and state; factors relating to the driver, such as behavior, characteristics and communication; and factors relating to the environment, such as social factors, demographics, characteristics and state.

**Figure 15:**
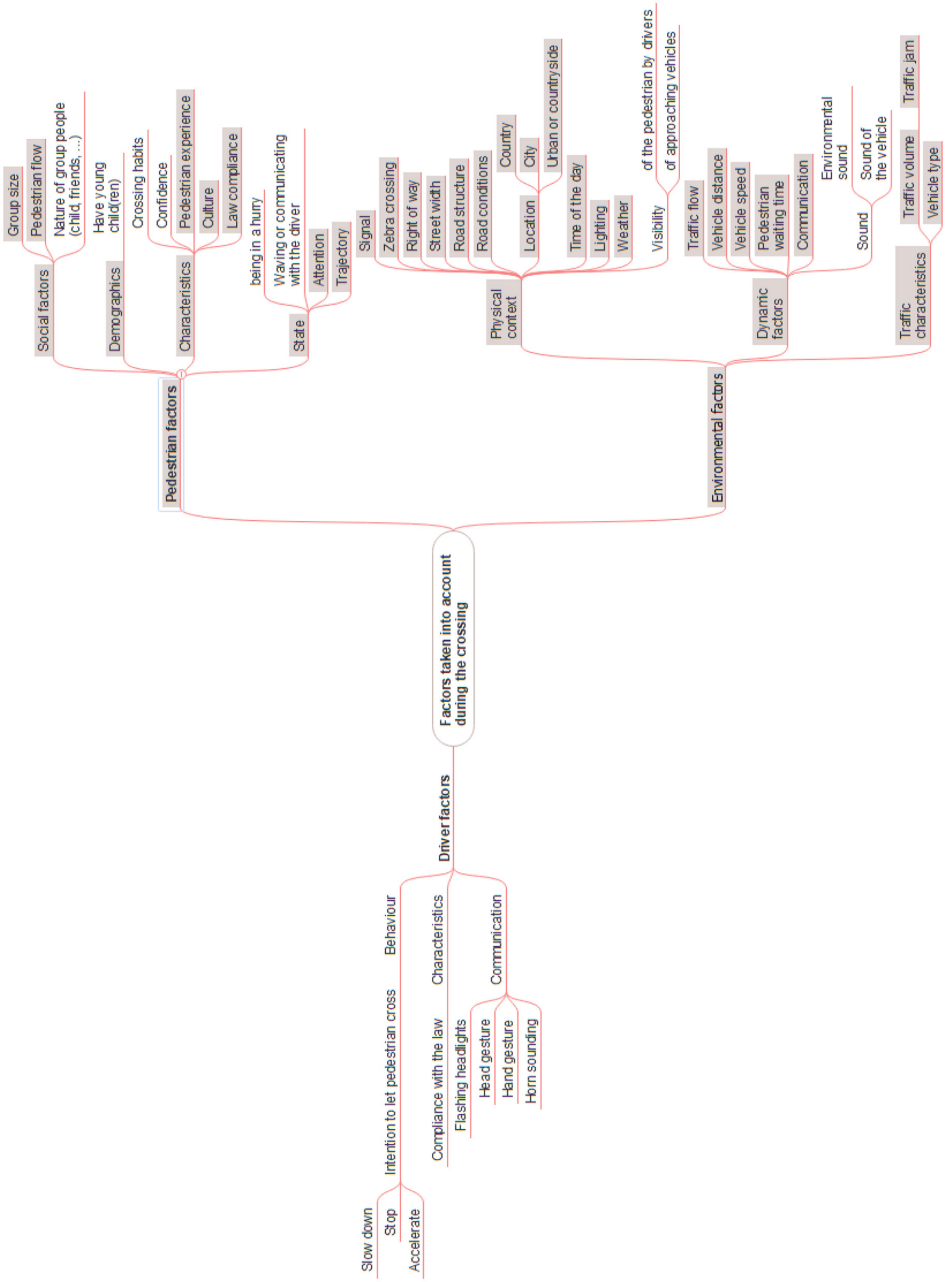
Factors considered when crossing the road (without FAVs): results from the first focus group. Factors also considered by Rasouli and Tsotsos (2019) are in grey. This image was manually produced.

During the first focus group, participants indicated the key factors that they considered when crossing the road in front of an approaching vehicle driven by a human. It is interested to see that they cited many of the same factors as Rasouli and Tsotsos (2019), represented in grey, in Figure 15. However, the participants also cited factors related to the driver, which were not considered by Rasouli and Tsotsos (2019).

Participants report taking into account **other pedestrians-related factors**, such as social, demographic, characteristic, and state factors, as in the model of Rasouli and Tsotsos (2019). They complete this model. They emphasize the importance of the nature of the pedestrian group to which they belong. Indeed, they will not act in the same way with a group composed of friends (they will tend to wait for each other) or with children (for example, some will force themselves to wait at the red light to set a good example). This shows that they are attentive to the demographic characteristics of the group of pedestrians waiting with them to cross the street. Regarding the dimension of the characteristics, they will modify their behavior according to their self-confidence, but also according to the trust they have in their environment, and according to their crossing habits in this particular space. Indeed, in a familiar space, the usual traffic patterns and driving style on that street are known, and as a result, they rely more on habits than on their senses. They are also attentive to their own state. If they are in a hurry, they will tend to make riskier decisions to cross. And if they communicate with the driver, with, for example, with a waving gesture, they will be more confident about stopping.

Regarding **environmental factors**, and particularly the physical context, the only change from Rasouli and Tsotsos (2019), is the emphasis on their own visibility (for example, they could be hidden by a billboard, or their clothing could impact their visibility), and that of the vehicle (for example, any street furniture that could obscure approaching vehicles). Participants emphasized sound as an important dynamic factor: the noise of vehicles gives them an indication of their position and speed. It was often mentioned during the discussions that participants listen before looking, whenever possible. Surrounding noise is also important (for example, construction work) because it can prevent them from hearing approaching vehicles.

The added dimension to the Rasouli and Tsotsos (2019) model, is the **driver factors**. Participants to the focus groups consider the driver's behavior (if they slow down or not or even accelerate), and the driver's characteristics, like it compliance with the law. Indeed, if as pedestrians they have observed that this driver is not respecting the highway code, for example by not stopping at a stop sign, then they will tend to be less likely to use the pedestrian crossing, even with a green light. The pedestrians consider also whether the driver try to communicate with them using the flashing lights (that means often I let you cross), head gesture (e.g., a slight nod to say I'll let you cross), hand gesture (e.g., invitation to cross or sorry I see you too late), and horn sounding (that often means do not cross, I pass first).

The added dimension to the model of Rasouli and Tsotsos (2019) lies in **driver-related factors**. Focus group participants took into account driver behavior (whether or not they slowed down, or even accelerated). It should be noted that for participants, it is the driver who accelerated, not the vehicle. They also placed importance on driver characteristics, such as their compliance with the law. Indeed, if they observed that this driver was not respecting the traffic rules (for example, by not stopping at a stop sign before the point where they wished to cross), then they would tend, even if the traffic light was green for pedestrians, to let the vehicle pass or wait until the last moment to cross (i.e., wait until the vehicle had stopped). Pedestrians also take into account whether the driver attempts to communicate with them by flashing their headlights (often meaning “I'll let you cross”), head gestures (e.g., a slight nod to say “I'll let you cross”), hand gestures (e.g., an invitation to cross or the “Sorry, I saw you too late” gesture), and honking horns (often meaning “Don't cross, I'll go first”).

From our study results, as pedestrians currently base part of their crossing decision on factors relating to the driver, it is difficult for them to consider how they would cross in front of a driverless vehicle. Therefore, different methods of communication are needed to replace this tendency of considering driver-related factors. Research is currently mainly focused on the use of eHMIs to create a communication channel between pedestrians and FAVs.

### 6.2 RQ2: What are the perceived benefits and risks or problems of FAVs?

From the first focus group, we note that pedestrians consider that the introduction of FAVs into the automotive landscape will have a great impact on both transportation and on society, with all the related advantages and disadvantages.

The main advantages cited are a decrease in accidents, improved traffic flow and public transport, better adaptation to specific audiences, a positive impact on ecology and for passengers, a transformation of society, and better recognition of and respect for emergency vehicles. Except the latter, all benefits cited by the participants are also found in the literature review. For example, previous studies have highlighted a potential reduction in accidents through better compliance with traffic rules (Harkin et al., 2024; Imprialou and Quddus, 2019), improved traffic flow and public transport through car-sharing and optimized mobility (Bagloee et al., 2016; Lu et al., 2020; Tirachini Hernández and Antoniou, 2020), ecological benefits such as lower greenhouse gas emissions and electric vehicle deployment (Greenblatt and Saxena, 2015; Jones and Leibowicz, 2019; Sanguesa et al., 2021), and advantages for specific audiences such as elderly or disabled people (Pettigrew, 2017). Passengers are also expected to benefit in terms of reduced stress and improved comfort (Pettigrew et al., 2018; Pettigrew and Cronin, 2019; Thomopoulos and Givoni, 2015). This means that, despite expressing the need for more information, the public is already well-informed about the research issues. The decrease in accidents is linked to better respect for traffic rules, as highlighted by Harkin et al. (2024).

Concerning the drawbacks, the participants cited accident hazards, computer and technological security risks, unsuitable road infrastructures, a negative ecological impact, lack of human interactions, lack of information for the public, transformation of society, increase in vehicle damage, and risks relating to the cohabitation of FAVs and human drivers. These concerns are consistent with issues highlighted in the literature. For instance, several studies have raised the question of technological reliability and vulnerability to hacking (Zanellato and Gombault, 2019), as well as the limitations of current infrastructures in supporting mixed traffic (Bagloee et al., 2016; Alsalman et al., 2021). Ecological drawbacks have also been discussed, particularly regarding the environmental cost of data storage and production of electrical components (Taiebat et al., 2018; Frieske and Stieler, 2022). The fear of reduced human interaction and increased social isolation has likewise been mentioned in previous work (Bissell et al., 2020). Finally, the challenge of coexistence between human-driven vehicles and FAVs has been identified as a major risk in early deployment phases, when heterogeneous traffic could create unpredictable dynamics (Rasouli and Tsotsos, 2019; Bonneviot et al., 2023).

### 6.3 RQ3: What could positively influence the acceptance of FAVs by pedestrians?

The participants considered that addressing the following aspects or questions could improve receptivity to FAVs and make them appear reliable: provide more information and assurance about the behavior of FAVs, thanks in particular to efficient eHMIs, provide suitable infrastructures, decrease transport costs, and define the legal and judicial framework. However, we can note that several research works focus on the legal aspect of FAVs (Contissa et al., 2017; Johnsen et al., 2017; Pattinson et al., 2020; Smith, 2013). It is therefore a question of (1) completing research on the subject, (2) implementing the recommendations resulting from the research, and finally (3) communicating the legal framework and rules to the general public.

The following research works address the question of designing effective eHMIs (Dey et al., 2018, 2020b,a; Eisma et al., 2019; Faas and Baumann, 2019; Faas et al., 2020; Holländer et al., 2019), adapting infrastructures (Alsalman et al., 2021; Bagloee et al., 2016; Tu et al., 2019), and the legal and ethical implications of introducing FAVs (Contissa et al., 2017; Johnsen et al., 2017; Pattinson et al., 2020; Smith, 2013).

We found only one paper that addressed the need for information (Liu et al., 2021). This work focuses on explaining the behavior and eHMI of an FAV when reacting to pedestrians and its positive impact. However, information is missing about how pedestrians feel about the FAVs, their behavior and eHMIs, as well as the legal framework and transformation of the infrastructures.

Informing pedestrians about the behavior and interface of FAVs, as well as the behavior expected of a pedestrian, is becoming a priority. To do this, we first need to identify which information needs to be communicated, in what form and by whom.

### 6.4 RQ4: How can communication take place between FAVs and a pedestrian wishing to cross the road?

The participants of the first focus group agreed that the FAV should communicate with pedestrians. Several communication methods were discussed. The participants considered whether the more comprehensive eHMIs could be based on existing signaling elements on the vehicle (e.g., headlights, horn, indicators). This is coherent with the results of (Tabone et al. 2023), who, using virtual reality, compared several ways to communicate to pedestrians that they could cross safely, ultimately preferring the one closest to existing infrastructures (Tabone et al., 2023). They also distinguish between the “normal" operating mode of an FAV and the “danger” mode, in the event that the FAV is not able to stop when it has detected a pedestrian trying to cross. In this case, the communication methods can be different, and the “danger" mode must be stronger in terms of signals emitted by the eHMI. For instance, if an eHMI displays a discrete green LED signal to indicate that the pedestrian can cross in normal mode, it could flash on a larger surface a red LED signal and honk the horn in danger mode.

However, to make sure that all pedestrians are able to receive the message and understand it, they ultimately proposed multiplying the communication methods for the same message (LED signals, sounds, text, icons, vibrating devices, etc.). It should be noted that, from the focus group participants' point of view, the eHMI text may be the least suitable for everyone, particularly for children, and those who are illiterate, partially sighted and blind, or people who are unable to read the local language. For these people, this method would cause a lot of confusion.

From both a safety and comprehensibility point of view, participants prefer the eHMI with projection on to the road. This preference is in line with recent findings by Sahaï et al. (2024), who tested projection-based and other eHMIs in a real-world track study with a communicating self-driving car. Their results showed that although the presence of such signals did not significantly increase pedestrians' self-reported feeling of safety, most participants were nevertheless able to interpret the meaning of the signals correctly or at least closely. Taken together, these results suggest that projection-based eHMIs are indeed comprehensible, supporting our participants' preference, but their effect on perceived safety is more limited and context-dependent. This highlights the importance of combining explicit projection cues with clear vehicle behavior in order to strengthen both understanding and the feeling of safety.

### 6.5 Limitations

This study has several limitations. First, the small number of participants (*N* = 14) necessarily restricts the generalizability of the findings. While focus groups are suitable for exploratory research, they are typically conducted with relatively small groups to encourage interaction and in-depth discussion (Cyr, 2019). In this respect, our study design is consistent with established methodological standards. Nevertheless, the results should be interpreted as qualitative insights rather than representative trends.

Second, all participants were French speakers, mostly from neighboring European countries. Cultural differences in pedestrian behavior, including communication norms and risk-taking tendencies (Rasouli and Tsotsos, 2019; Pelé et al., 2019), mean that the applicability of our findings to broader populations remains limited. Conducting the focus groups in French also implies that participants shared not only a common language but also cultural references and ways of reasoning about mobility, which may have shaped the types of concerns expressed. However, we also observed disparities within our sample, particularly between participants living in Luxembourg and those from other countries, suggesting that cultural or national context may already influence how pedestrians perceive and discuss interactions with autonomous vehicles. In contexts where different languages are spoken, or where road culture is less rule-oriented and more reliant on implicit negotiation, pedestrians may interpret or prioritize vehicle communication signals differently.

Third, the online setting of the focus groups may also have influenced participant dynamics and reactions. Although online platforms facilitate participation and allow for structured interactions, they can reduce the richness of non-verbal exchanges and potentially alter the way participants express concerns or react to visual materials compared to in-person settings.

Fourth, the age distribution was not sufficiently balanced. Prior work has shown that younger and older pedestrians may rely on different cues when deciding whether to cross in front of an automated vehicle (Sahaï et al., 2024). Younger participants often rely more on implicit indicators such as speed or trajectory, whereas older pedestrians tend to prefer explicit signals and require longer decision-making times. Since older adults were under-represented in our sample, our results may underestimate the importance of these explicit cues and overemphasize the role of implicit perception. In addition, no participant reported having a disability, which limits the transferability of our findings to populations for whom accessibility and safety concerns may be particularly salient. Although some participants were parents with young children, they remained a minority within the group, and issues specific to accompanying children, such as increased vigilance, teaching safe crossing behaviors, or concerns about visibility, were less frequently raised in the discussions.

Taken together, these limitations underline the need for future research with larger and more diverse samples, both in terms of cultural background and demographic groups, and conducted in varied settings, in order to validate and extend the present results.

## 7 Conclusion

The promised arrival of FAVS raises many questions, both in scientific research and among the general public. In two focus groups with 14 participants in total, we gathered pedestrians' concerns not only about the future society with the introduction of FAVs into the automotive landscape, but above all, their communication with FAVs in crossing situations.

In terms of the factors considered when crossing the road in front of a human-driven vehicle, they noted that they also considered factors relating to the driver. This explains the complexity of replacing this habit with electronic signals. However, with such a small and non-representative sample, these insights should be regarded as exploratory rather than generalizable findings. They highlight points of alignment with previous research but cannot support broad claims that “researchers and the public are largely in agreement.”

When it comes to the advantages and disadvantages of FAVs, participants expressed both expectations and concerns. They mentioned benefits such as improved safety, smoother traffic flow, and better adaptation to specific audiences, but also underlined unresolved challenges, including the difficulty of addressing specific pedestrians in groups, the risk of reduced human cues, and the complexity of ensuring inclusive and accessible communication systems. Importantly, these challenges cannot be solved by a single technical fix. For instance, designing an eHMI able to indicate clearly which pedestrian is targeted in a group situation raises questions of signal clarity, potential ambiguity, and even fairness when several pedestrians intend to cross simultaneously. Similarly, multimodal communication strategies (visual, auditory, and haptic) may improve inclusivity but also increase risks of overload or misinterpretation. These considerations highlight that technical feasibility and social acceptability must be jointly addressed.

Concerning the elements needed to foster the acceptance of FAVs by the public, the focus group participants expressed the need for more information about FAVs, their behaviors and how they will be introduced into the automotive landscape. These findings resonate with acceptance models such as TAM and UTAUT, where perceived usefulness, trust, and social influence play a key role. The participants' emphasis on transparency, trialability and opportunities to observe or test FAVs also echoes extensions of these frameworks, suggesting that adoption will depend on more than interface design alone.

In terms of eHMIs, the focus group participants preferred one that projects a green pedestrian crossing onto the road to indicate that the FAV will wait for the pedestrian to cross. However, they also proposed reusing the existing signalisation systems on vehicles to communicate with pedestrians. Participants also imagined broader urban transformations, such as shared spaces without pavements, which underline the societal scope of the changes anticipated. At the same time, these visions point to how much remains to be explored beyond confirming known preferences for visual communication.

It could be interesting to repeat these focus groups with other target groups (i.e., young people, children, the elderly, disabled people, visually impaired or color-blind individuals and illiterate people, as well as those who do not speak the language used). The eHMIs presented to the participants should be tested by a higher number of people, and in real or simulated situations. In addition, further research should be carried out to precisely identify the type of information required, the nature of this information and the entities that should provide it.

## Data Availability

The datasets presented in this article are not readily available because the data have been destroyed to correspond to the signed consent form. Requests to access the datasets should be directed to lou.schwartz@list.lu.

## Footnotes

1 Pedestrian Traffic Fatalities by State: 2022 Preliminary Data (January–December), Governors Highway Safety Association, 2023 https://ghsa.org/resource-hub/pedestrian-traffic-fatalities-state-2022-preliminary-data-january-december.

2 Preliminary Statement of Policy Concerning Automated Vehicles, National Highway Traffic Safety Administration (NHTSA), 2013 https://www.nhtsa.gov/sites/nhtsa.gov/files/documents/automated_vehicles_policy.pdf.

3 SAE Levels of Driving Automation Refined for Clarity and International Audience, SAE International (SAE), 2021, https://www.sae.org/blog/sae-j3016-update.

4 The Smiling Car, 2022 https://semcon.com/smilingcar/.

5 Jaguar Land Rover's virtual eyes look at trust in self-driving cars, 2018 https://media.jaguarlandrover.com/news/2018/08/jaguar-land-rovers-virtual-eyes-look-trust-self-driving-cars.

6 Autonomous concept car smart vision EQ fortwo: Welcome to the future of car sharing, Mercedes-Benz Media, 2017 https://media.mercedes-benz.com/article/057a0a95-4911-4f9b-913d-d96f902b1132.

7 Mitsubishi Electric Introduces Road-illuminating Directional Indicators, 2015 https://www.mitsubishielectric.com/news/2015/1023_print.html.

8 World premiere of the Mercedes-Benz F 015: Luxury in Motion at the CES, Mercedes-Benz Media, 2015 https://media.mercedes-benz.com/article/3c2731cc-bc26-4bbb-968e-3672b76ba76d.

9 Microsoft Teams, 2025,https://www.microsoft.com/en-us/microsoft-teams/group-chat-software.

10 Miro, 2025, https://miro.com/.
